# Unravelling the hidden diversity of cave mycobiota in Thailand’s Satun Geopark

**DOI:** 10.1038/s41598-023-43316-2

**Published:** 2023-11-06

**Authors:** Satinee Suetrong, Sita Preedanon, Noppol Kobmoo, Charisa Srihom, Sayanh Somrithipol, Supicha Saengkaewsuk, Prasert Srikitikulchai, Anupong Klaysuban, Salilaporn Nuankaew, Charuwan Chuaseeharonnachai, Boonchuai Chainuwong, Chotika Muangsong, Kittapha Malimart, Nattawut Rungjindamai, Chaiyaporn Siripornpibul, Umapon Chareonkunnatum, Bumrungrat Ploydam, Narongrit Thungprue, Sissades Tongsima, Zhi-Feng Zhang, Lei Cai, Nattawut Boonyuen

**Affiliations:** 1 National Center for Genetic Engineering and Biotechnology (BIOTEC), National Science and Technology Development Agency (NSTDA), Khlong Nueng, Khlong Luang, 12120 Pathum Thani Thailand; 2 Innovation for Social and Environmental Management, Mahidol University (MU), Amnatcharoen Campus, Amnatcharoen, 37000 Thailand; 3 Department of Biology, School of Science, King Mongkut’s Institute of Technology Ladkrabang (KMITL), Bangkok, 10520 Thailand; 4 Department of Groundwater Resources, Ngamwongwan 54 Lat Yao, Chatuchak, Bangkok, 10900 Thailand; 5 Department of Mineral Resources, Region 4, Tha Kham, Phunphin, Surat Thani, 84130 Thailand; 6 Khao Banthat Wildlife Sanctuary, Ban Na, Srinagarindra District, 93000 Phatthalung Thailand; 7 Thung Wa, Thung Wa District, 91120 Satun Thailand; 8 Southern Marine Science and Engineering Guangdong Laboratory (Guangzhou), Guangzhou, 51145 China; 9 State Key Laboratory of Mycology, Institute of Microbiology, Chinese Academy of Sciences (CAS), Beijing, 100101 China

**Keywords:** Fungi, Ecology, Evolution, Microbiology

## Abstract

Karst caves are distinctive ecosystems that have limited nutrients, darkness, low to moderate temperatures, and high moisture levels, which allow for a diverse range of fungal communities to thrive. Despite their significance, little is understood about the fungi found in karst caves in Thailand. In 2019, we studied the cultured mycobiota from five substrate types (air, water, rock, soil/sediment, and organic debris) in two karst caves (Le Stegodon and Phu Pha Phet Caves) of the Satun UNESCO Global Geopark, southern Thailand. A cumulative count of 829 distinct fungal morphological types was identified, encompassing 319 fungal culturable were observed. Based on preliminary analyses of the internal transcribed spacer (ITS) sequence using BLAST searches, the most common phylum among the fungal morphotypes was Ascomycota, harboring 282 species in 91 genera, 93.4% of which were distributed in the classes Eurotiomycetes, Sordariomycetes, and Dothideomycetes. The most common fungal genera identified in the two karst caves were *Aspergillus, Penicillium, Cladosporium, Talaromyces, Xylaria*, and *Trichoderma*, with 45, 41, 24, 14, 14, and 6 species identified, respectively. Discovering fungi in Thai karst caves highlights the extensive fungal diversity in the Satun UNESCO Global Geopark, implying undiscovered species, and emphasizing the need for comprehensive investigations in other unexplored Thai karst caves.

## Introduction

Fungi exhibit a diverse range of physical, environmental, metabolic, and evolutionary characteristics and play significant roles in ecosystems as decomposers, mutualistic partners, and disease-causing agents^1–3^. Despite estimates of fungal diversity ranging from 2.2 to 3.8 million species, 96% of fungal species remain unknown^4^. Fungi in caves have adapted to low nutrient availability, darkness, coolness, and humidity, making them unique environments for studying extreme-adapted fungi^5,6^. Karst cave fungi are essential in biogeochemical cycling, speleothem formation, and the development of caves^7,8^.

Cave fungi interact with minerals, fix nitrogen, and utilize aromatic compounds, influencing fungal metabolism and cave biogeochemistry^7^, and potentially producing novel compounds, bioactive secondary metabolites, and/or enzymes, enabling them to survive in harsh environments^9,10^. Most cave fungi are not native to caves and are likely introduced by humans, water, air currents, and animals, with Ascomycota being the most common phylum^11–13^. Recent studies in various caves worldwide have identified high fungal diversity and numerous new taxa across various substrates^14–17^.

Nuankaew et al.^18^ recently discovered two new *Talaromyces* species in soil samples from the Satun UNESCO Global Geopark in Thailand, a region with scarce documentation of fungi in tropical caves. As part of a mycological diversity study in the Satun UNESCO Global Geopark, this research aimed to explore the fungi present in two karst caves, Phu Pha Phet Cave and Le Stegodon Cave. In the research question of the study, how does the fungal diversity differ between Phu Pha Phet Cave and Le Stegodon Cave in the Satun UNESCO Global Geopark region, given their distinct environmental characteristics, particularly when compared against each other? what are the fungal classifications, levels of mycological taxonomy, and various types of cave fungi discerned from diverse substrates such as air, water, rock, soil/sediment, and organic matter, employing techniques like culturable morphotypes and the BLAST tool available in NCBI? The primary objective of this research is to explore and identify the fungal diversity in two specific karst caves within the Satun UNESCO Global Geopark region. By examining culturable morphotypes and employing BLAST queries against a fungal sequence database, the study aims to evaluate and compare the taxonomic classifications and diversity of fungi isolated from different sources, including air, water, rock, soil, and organic debris substrates within two distinct caves.

## Materials and methods

### Cave information and sampling sites

Satun Province in southern Thailand, recognized as the "Land of Palaeozoic fossils," became the country's first UNESCO Global Geopark on April 17, 2018, according to Cheablam et al.^19^. The Satun UNESCO Global Geopark encompasses four districts in Satun Province, known for diverse karst topography and abundant fossils from the Palaeozoic Era. Phu Pha Phet Cave, or "Diamond Mountain Cave," in Satun Province, Thailand, is the country's largest and the world's fourth-largest cave, with a length of 536.65 m and an area of 0.16 hectares. The cave has over 20 chambers with dazzling stalactites and stalagmites resembling diamond flakes, accessed via a wooden bridge and illuminated by lighting. Tourist visits are allowed with restricted access for conservation and sustainable tourism purposes, resulting in limited visitation and moderate human interference^20^.

**Figure 1:**
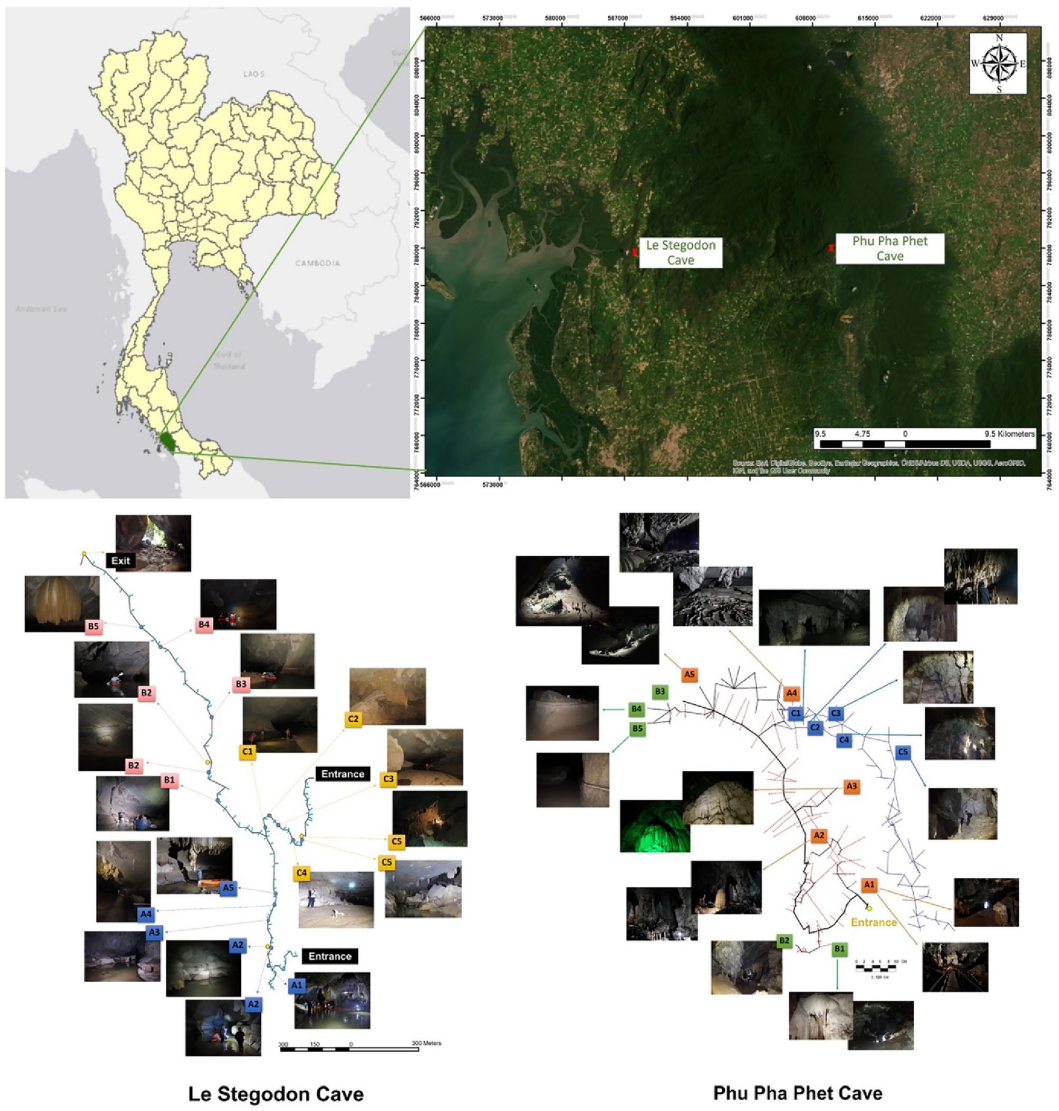
Locations of the two sampling sites. Map of Thailand and location of the Satun UNESCO Global Geopark in Satun Province in the southern part of Thailand. Sampling sites/zones (A1–A5), (B1–B5) and (**C**) (C1–C5) in Le Stegodon Cave are shown, as are the locations of sampling sites/zones (A1–A5), (B1–B5) and (C1–C5) in Phu Pha Phet Cave. The maps of caves were modified from Siripattharapurinont^21,22^ and Muangsong et al.^23^. The map was produced using World Imagery from (https://www.arcgis.com/home/) and the Light Gray Canvas Map via ESRI ArcGIS Desktop 10.5.1. (https://livingatlas-dcdev.opendata.arcgis.com/maps/esri::light-gray-canvas-1/about; https://desktop.arcgis.com/en/system-requirements).

Le Stegodon Cave in Thung Wa Subdistrict of Satun Province is a 107 m high sea cave with 3 winding tunnels converging within the mountain. It spans 3–4 km with tunnel widths/heights of 10–20 m and seawater intrusions at the westernoutlet (Fig. 1). Cave's western outlet links to a brackish stream via mangrove forest, affected by daily tides. Restricted areas exist for tourists, similar to Phu Pha Phet Cave, with low to moderate human interference. Both caves were divided into three zones for fungal sampling^20^.

### Sample collection

In November 2019, 64 cave samples were collected; 30 from Le Stegodon Cave and 34 from Phu Pha Phet Cave. Phu Pha Phet Cave has two main zones (A and B), each with five subsections (A1–A5 and B1–B5), situated along the main cave pathway. The cave can be accessed by a wooden bridge (Fig. 1). Zone A (A1–A5) shows more human disturbance than Zone B in Phu Pha Phet Cave. Zone C is distinct from the main chamber, has flowing water, and is free from human activity due to visitor restrictions (Fig. 1).

Le Stegodon Cave's Zone A (A1–A5) is close to the entrance and has high tourist traffic. Zone B (B1–B5) has minimal human interference, separated from Zone A's main chamber/route, allowing for outside air circulation. Zone C (C1–C5) is isolated from the main chamber and inaccessible to tourists, resulting in no human disturbance (Fig. 1). Five types of samples (air (A), water from stalactites (W), soil or sediment (S), organic litter (O), and rock (R)) were randomly collected according to the method of Zhang et al.^5,12^. Per cave zone, we obtained 5 A, 1 W, 1 S, and 1 R samples. Mixed five randomly collected subsamples per type in water and soil sampling, forming one sample per type. For the rock sampling, one rock was collected from each zone of both caves. Six and ten organic samples were collected from Phu Pha Phet Cave and Le Stegodon Cave, respectively. Thus, a total of 34 samples were collected from Phu Pha Phet Cave (5 A, 1 W, 1 S, 1 R per zone and 6 organic samples), and 30 samples were collected from Le Stegodon Cave (5 A, 1 W, 1 S, 1 R per zone and 10 organic samples). The collection and isolation methods were specific to the type of sample collected. Air (A) samples were collected using the Koch sedimentation method^5^, while water (W) samples were collected in 10 mL subsamples and stored at 4 °C in 50 mL sterile centrifuge tubes. Soil (S) subsamples of 10–20 g were taken and stored in zip-lock bags at 4 °C, and rock (R) samples were randomly collected and stored in zip-lock bags at 4 °C until isolation according to the method of Ruibal et al.^24^. Organic litter samples (O) were collected in plastic zip-lock bags and stored at 4 °C. All samples were transferred to the mycological laboratory and kept at 4 °C in a refrigerator until fungal isolation was performed^25^. Physical properties of cave environments and water samples were recorded and analysed at the Mineral Resources Analysis and Research division of the Thailand Department of Mineral Resources. Rock samples (R) were also analysed at the same facility (Table S1).

### Isolation of cave fungi and culture collection

**Figure 2:**
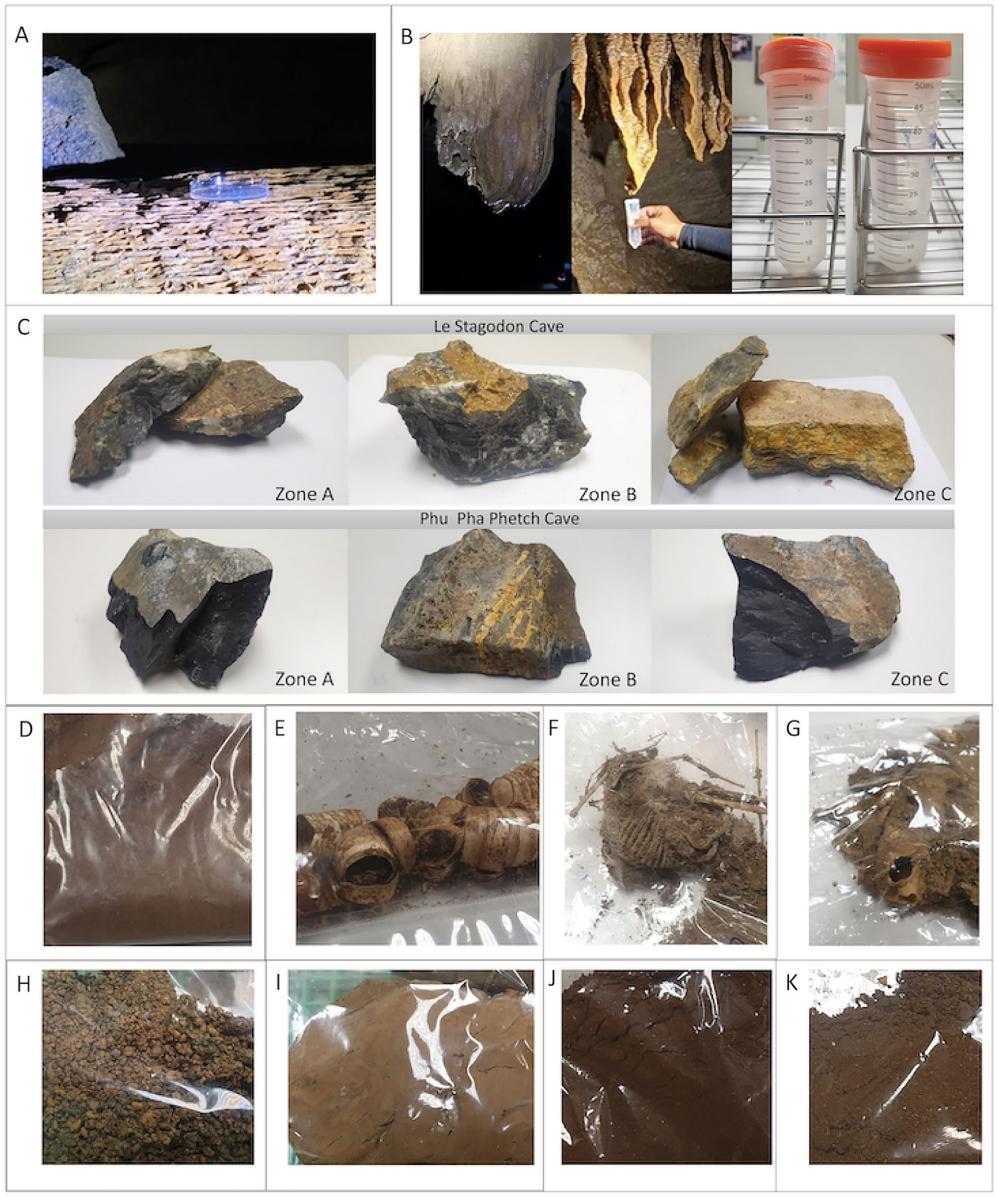
Five different types of substrates used for collecting cave fungi. These include (**A**) atmospheric samples (air), (**B**) water samples, (**C**) rock specimens from three separate zones, (**D**–**H**) collections from organic debris, and (**H**–**K**) soil or sediment samples. These were collected from two locations, Phu Pha Phet and Le Stegodon Caves.

Water (W), soil (S) and organic litter (O) samples (Fig. 2) were processed according to the methods described by Zhang et al.^5^. One gram of soil or organic litter or 1 mL of a water sample was added to 9 mL of sterile water in a sterile 15-mL centrifuge tube, and the tube was shaken by hand or mechanically for 10 min. The suspensions of water and soil samples were diluted to 10^–1^–10^–5^ and 10^–2^–10^–5^ for organic litter. Two hundred microliters of suspension at each concentration were spread on PDA (potato dextrose agar) plates containing streptomycin (50 µg/mL) and ampicillin (50 µg/mL); three replicate plates were prepared for each dilution.

Rock (R) samples and limestones were washed with 95% ethanol for 5–7 min to remove contamination with dust and airborne spores, and then once with sterile water containing 0.1% Tween 20. The rocks were ground into powder using a sterilized mortar and pestle, and suspensions were made by adding the ground samples to sterilized water at concentrations of 10^–1^ to 10^–2^ according to previously described methods^24,26^. Three PDA plates supplemented with ampicillin (50 µg/mL) and streptomycin (50 µg/mL) were used to place rock powder suspensions of 300, 500, and 1000 µL. Fungal plates were incubated at room temperature (23–25 °C) and examined at 24-h intervals for 2–3 weeks, and single colonies were picked from the plates and used to inoculate new PDA plates without antibiotics. Fungi cultures were maintained on PDA slant agar plates with mycelium or spore suspensions. Fungal strains were selected by morphotypes, stored at −80 °C with the cryopreservation method. Fungal cultures were kept in the Thailand Bioresource Research Center (TBRC; https://www.tbrcnetwork.org) and the National Biobank of Thailand (NBT; https://www.nationalbiobank.in.th), and dry cultures were deposited at the BIOTEC Bangkok Herbarium (BBH; https://www.nationalbiobank.in.th/microbe-services).

### Molecular studies

Total genomic DNA was extracted from 7-day-old axenic cultures grown on PDA at room temperature according to the modified method of Boonyuen et al.^27^. Internal transcribed spacers 1 and 2, including the intervening 5.8S nrDNA gene (ITS), were sequenced for every fungal isolate^28^. PCR was performed in a 50-µL reaction mixture containing 35.8 µL of nanopurified water, 5 µL of 10X Taq buffer with (NH_4_)2SO_4_, 5 µL of 25 mM Mg_2_Cl, 1 µL of 10 mM dNTP, 1 µL of each primer, 0.2 µL of recombinant Taq DNA polymerase (Thermo Scientific™) and 1 µL of fungal DNA. The primers ITS5/ITS4, ITS1F/4, NS1/NS4 were employed^28^. PCR profiles were obtained using the T100™ Thermal Cycler (BIO–RAD Laboratories, Inc., California) as described by Zhang et al.^5,12^. Electrophoresis was performed on a 1% agarose gel with a known-size DNA ladder to check PCR products. MACHEREY–NAGEL's DNA purification kit was used to confirm amplicon presence, followed by direct DNA sequencing. Sequencing was carried out at Macrogen Inc. (South Korea) using the primers listed above. Paired forward and reverse reads were used to generate consensus sequences, and BioEdit 7.2.5^29^ was used to check for ambiguous bases and trim sequences at both ends. A BLAST search of the GenBank database for the generated sequences was performed to exclude contamination and to search for related taxa (www.ncbi.nlm.nih.gov/blast; accessed 27 February 2022). Sequence homologies were analyzed using the BLAST search engine at NCBI and compared with the sequences reported in GenBank.

### Diversity analysis

Due to the large number of morphotypes obtained in this study (829), we were unable to molecularly identify all morphotypes using multiple markers. In addition to the strains that could be identified to the species level using ITS, only strains that represented candidates for new species were thoroughly examined using multiple markers^5,12^. In this study, initial identification of fungal morphotypes was based on their cultural characteristics, and a more detailed species-level identification incorporated both morphotype-based identification and ITS-DNA sequence data. Examination of the nucleotide sequences revealed high similarity, approximately 91.63–99.53%, among strains within the same morphotype. The ITS and 5.8S sequences from species across various genera were procured from GenBank to assess the nucleotide similarity both within and among well-established genera and species. The legitimacy of these 'morphotypes' as taxonomic groups was confirmed through ribosomal DNA sequence analysis. As a result, this study employed the notion of fungal species defined by morphotypes and ITS data to investigate the comprehensive diversity of the fungal community. For all morphotypes, colonies recorded from all samples, dilutions and replicates were counted as abundance data for the respective samples. The data were used to infer species richness, rarefied species richness, Shannon’s diversity index, and Simpson’s diversity index for each sample using the ‘vegan’ package in R^30^. We tested the effects of differences in the cave and zone from which the sample was obtained and the effect of differences in the type of sample, as well as the effects of interaction among these factors, on Simpson’s diversity index via ANOVA in a linear model using cave, zone and sample type as fixed effects. The per-sample diversity as calculated above informs us about the diversity we might find, on average, per sample. To gain further insight into the overall diversity of the respective caves (Phu Pha Phet and Le Stegodon), sample types (air, organic litter, rock, soil and water) and sampling zones (A, B and C), the samples were pooled into corresponding categories, and the diversity indices were recalculated. Species accumulation curves were obtained using the rarefaction method and extrapolation with the Hill number^31^ using the iNEXT package in R^32^.

## Results

### Physical–chemical data from Phu Pha Phet and Le Stegodon Caves

The Phu Pha Phet Cave had low light intensity except at location A5, which had an opening in the ceiling allowing natural light in. The air temperature was 25.1 °C on average, and rock surface temperatures ranged from 22.7 to 26.5 °C. Carbon dioxide (CO_2_) concentrations ranged from 407 to 839 ppm, with an average of 547 ppm. Relative humidity was 99.9% in Zones A, B, and C, with Zone A being a primary tourism route. Air and rock temperatures in Zone A ranged from 24.8 to 27.3 °C and 24.9 to 26.4 °C, respectively. The highest CO_2_ concentrations were recorded at A4 and A5 due to their proximity to an underground river, cave pools, and a cave window. Zone C had limited air circulation, diverse mineral formations, and elevated CO_2_ concentrations ranging from 555 to 839 ppm, with an air temperature of 24.9 to 25.3 °C (Table S1).

During the survey period, the Le Stegodon Cave was dark with low light intensity. The relative humidity ranged from 99.1 to 99.9%, with an average of 99.8%. Average temperatures during the survey were 25.8 °C for the air and 25.7 °C for the rock surfaces. Cave air circulation was good, and CO_2_ concentrations were generally normal (average 556 ppm, range 431–669 ppm) due to ventilation, although deeper areas had higher concentrations (C1: 669 ppm, C3: 630 ppm, C4: 607 ppm) likely due to streams and active stalagmites/stalactites. Underground stream water had a pH of 7.4, an electrical conductivity of 188 µS/cm, salinity of 1.18 ppt, and dissolved oxygen of 6.02 mg/L. Drip water in Zone C had a calcium carbonate concentration ranging from 196 to 321 mg/L, with an average of 250 mg/L (Table S1).

### Diversity of karst cave-dwelling fungi

**Figure 3:**
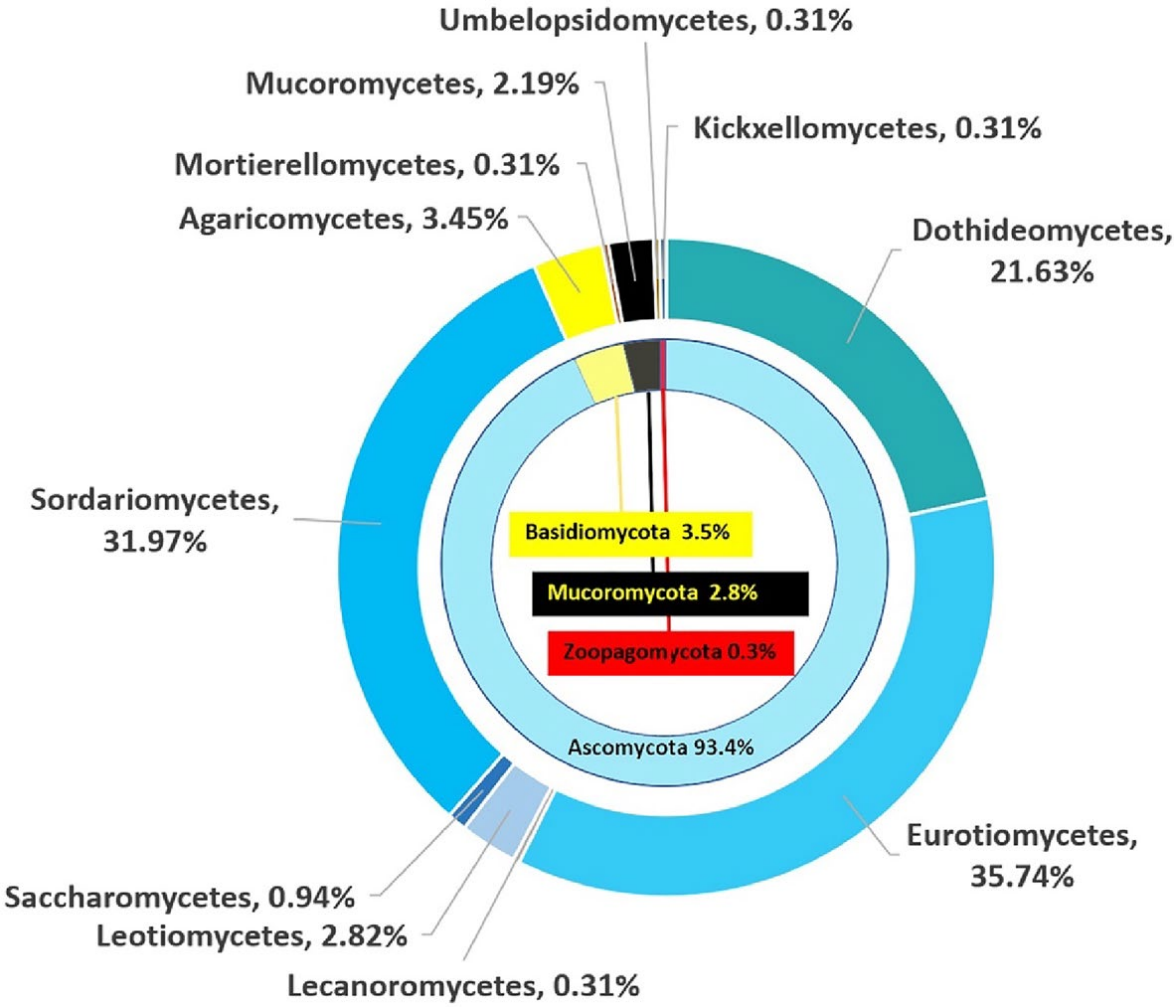
An overview of the 319 fungal culturable isolates, arranged according to class and phylum, obtained from the two caves; the percentage of fungal isolates belonging to each classification is shown.

**Figure 4:**
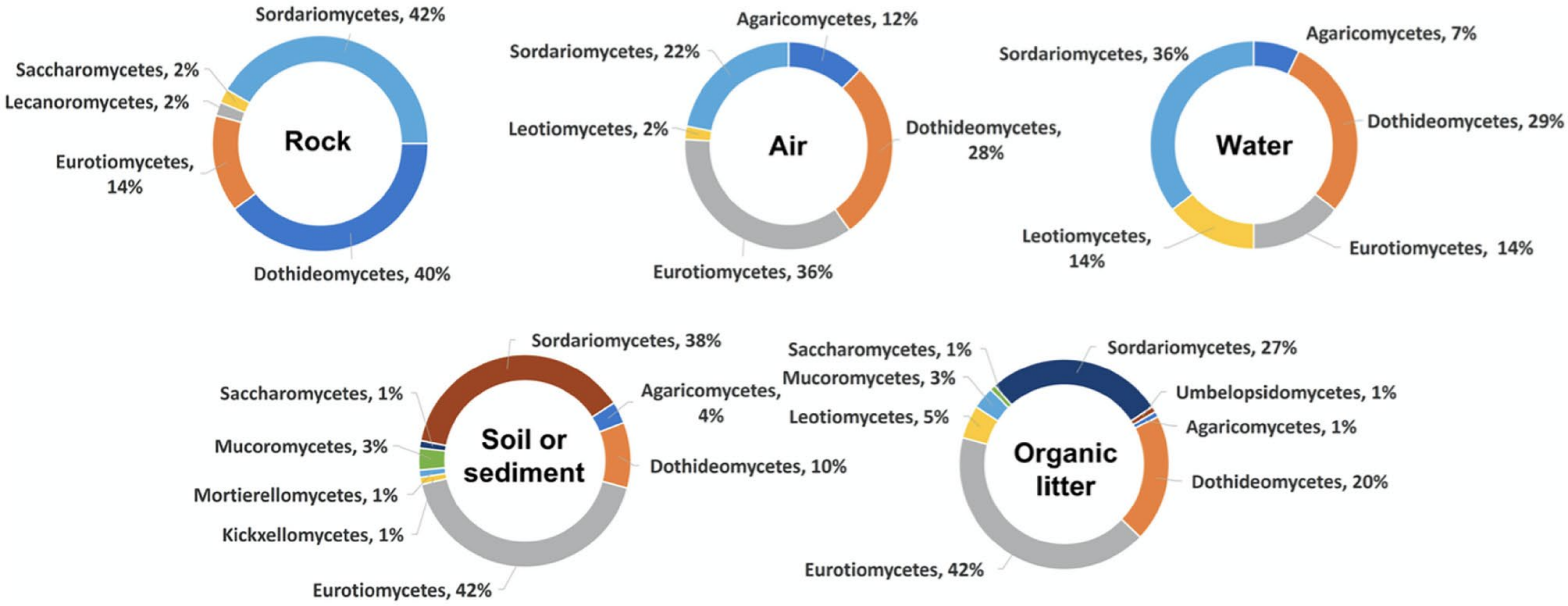
Percentages of fungal isolates obtained from the five different types of specimens (rock, air, water, soil/sediment and organic litter) collected from the two karst caves (Phu Pha Phet and Le Stegodon Caves) shown at the class level.

Figure 3 presents an overview of 319 culturable fungal isolates obtained from the Le Stegodon and Phu Pha Phet Caves. The isolates are classified based on their taxonomic classifications within the fungal kingdom. Thirty-four fungal samples were collected from Phu Pha Phet Cave, and 30 fungal samples were collected from Le Stegodon Cave. Samples of air, water, rock, soil/sediment, and organic litter yielded 1145 and 823 fungal isolates from Phu Pha Phet Cave and Le Stegodon Cave, respectively. Of these, 829 different morphotypes in 319 culturable isolates were preliminarily classified using a BLASTn search in NCBI based on ITS data. Among the 319 culturable isolates, Ascomycota was the most common phylum, with 282 species in 91 genera, representing 93.4% of the total species. The remaining isolates were distributed among Basidiomycota (3.5%), Mucoromycota (2.8%), and Zoopagomycota (0.3%), as displayed in Fig. 3. Within Ascomycota, the most abundant classes were Eurotiomycetes, Sordariomycetes, and Dothideomycetes, and this pattern was consistent across all samples and in each individual cave (Figs. 3 and 4; Figs. S1–S3).

In the context of this research, we identified the three most frequently observed categories of fungi in two caves. These fell under the taxonomic classes known as Eurotiomycetes, Sordariomycetes, and Dothideomycetes, as shown in Fig. 4 and Figs. S1–S3. These classes represent a broad diversity of fungal life, each with unique characteristics and ecological roles within cave systems.

**Figure 5:**
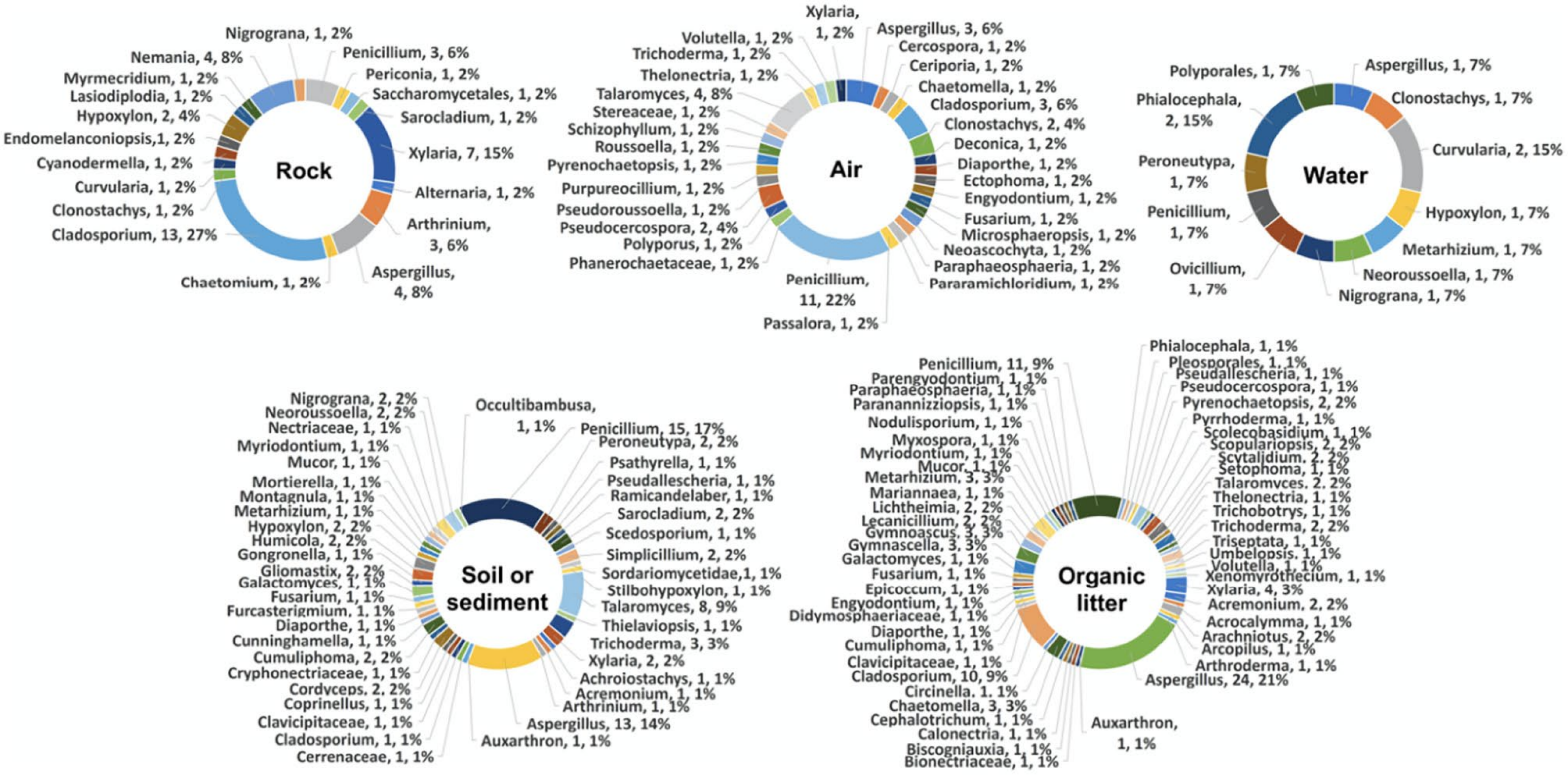
Percentages of fungal isolates obtained from the five different types of specimens (rock, air, water, soil/sediment and organic litter) collected from the two karst caves (Phu Pha Phet and Le Stegodon Caves) shown at the genus level.

Nineteen genera (46 species), 31 genera (50), 12 genera (14), 44 genera (90), and 55 genera (108) were obtained from the air, water, rock, soil/sediment, and organic litter samples, respectively, that were collected, indicating a high degree of mycological diversity in two karst caves (Fig. 5). The most common fungal genera obtained from the samples were *Aspergillus, Penicillium, Cladosporium, Talaromyces, Xylaria,* and *Trichoderma,* with 45, 41, 24, 14, 14 and 6 species, respectively (Fig. 5, Table S2)*.*

As shown in Figs. S4 and S5, the most species-rich orders observed in the rock samples found in Le Stegodon Cave and Phu Pha Phet Cave were Xylariales (50%) and Cladosporiales (56%), respectively. The most frequently observed orders documented from the air samples obtained from Le Stegodon Cave were Pleosporales and Hypocreales (22%), and Eurotiales (47%) was the order most frequently found in the air samples from Phu Pha Phet Cave. Hypocreales and Pleosporales (33%) and Eurotiales and Pleosporales (25%) were the most common fungal orders found in the water samples from Le Stegodon Cave and Phu Pha Phet Cave, respectively. In the soil/sediment samples from both caves, Eurotiales was the dominant order, accounting for 44% and 36%, respectively, of the obtained species. In the organic litter samples from Le Stegodon Cave, the most abundant fungal order was Hypocreales (23%), with 25 species obtained, while the most abundant order in the organic litter samples from Phu Pha Phet Cave was Eurotiales (41%).

**Figure 6:**
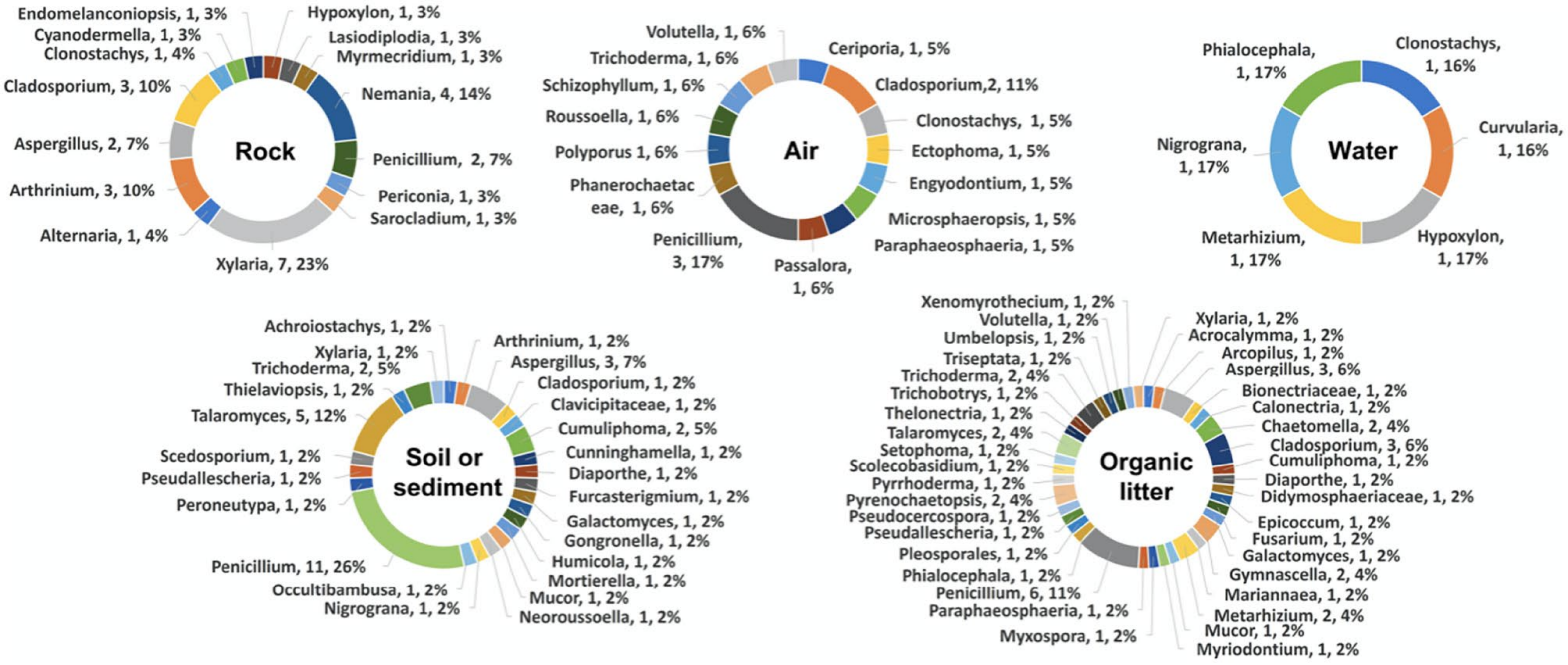
Percentages of fungal isolates obtained from 5 different fungal specimens (rock, air, water, soil/sediment and organic litter) from Le Stegodon Cave calculated at the genus level.

Table S2 and Fig. 6 revealed that *Penicillium* was the most frequently isolated genus from air, soil, and organic litter in Le Stegodon Cave, whereas *Xylaria* dominated in rock samples as shown in Fig. 6. In water samples, the four most common genera in Le Stegodon Cave were *Hypoxylon, Nigrogana, Metralizium*, and *Phialocephala* (Fig. 6).

**Figure 7:**
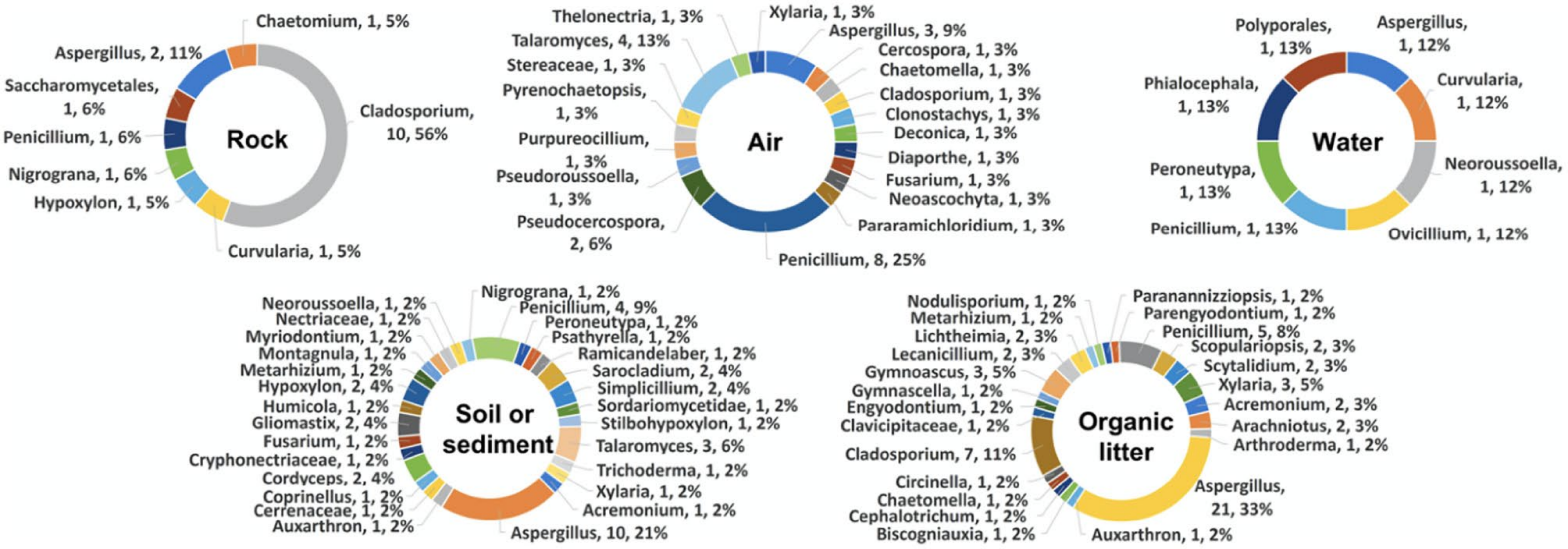
Percentages of fungal isolates obtained from 5 different fungal specimens (rock, air, water, soil/sediment and organic litter) from Phu Pha Phet Cave calculated at the genus level.

In Phu Pha Phet Cave, *Cladosporium, Penicillium, Aspergillus*, and *Aspergillus* were the most common fungal genera in rock, air, soil/sediment, and organic litter, respectively. *Penicillium, Talaromyces*, and *Aspergillus* were the top three most common genera found in air samples from Phu Pha Phet Cave (Fig. 7).

The study found diverse fungal species in Le Stegodon Cave's three zones (A–C), with Zone A having 31 species and 17 fungal groups, Zone B having 32 species and 26 genera, and Zone C having 30 species and 18 genera. Ascomycota was the dominant phylum across all zones, with *Penicillium* being the most common genus (Fig. S6). Mucoromycota and Basidiomycota were also present. Zone A had *Gongronella butleri, Mortierella capitata, Polyporus arcularius,* and *Phanerochaetaceae* sp., Zone B had *Cunninghamella binariae* and *Ceriporia lacerata*, and Zone C had *Mucor* sp. and *Schizophyllum commune*. The specific areas designated A and B had fungi belonging to Ascomycota, Basidiomycota, and Mucoromycota. Le Stegodon Cave is renowned for its rich fungal biodiversity.

Zone C is considered a separate hall and is not accessible to tourists, and a different set of microbes were found there. As shown in Figure S7, the diversity of the fungi in Phu Pha Phet Cave differed among zones A-C within the cave. The results revealed that Zone A contained 16 genera and 33 common fungal species, the majority of which belong to the phylum Ascomycota, particularly the genera *Aspergillus* (24%), *Talaromyces* (15%), *Hypoxylon, Penicillium* (9%), *Curvularia*, and *Xylaria* (6%). Zone B contained 21 genera and 38 common species, the majority of which belong to the phylum *Ascomycota*, particularly the genera *Aspergillus* (18%), *Penicillium* (16%), and *Cladosporium* (8%). The *Basidiomycota* was represented by *Coprinellus disseminates, Deconica* sp., *Polyporales* sp., *Psathyrella pygmaea* and *Stereaceae* sp. Finally, Zone C contained 20 genera and 32 common fungal species, the majority of which belong to the phylum Ascomycota, specifically the genera *Cladosporium* (19%), *Penicillium* (16%), *Cordyceps, Fusarium,* and *Gliomastix* (6%). The Zoopagomycota was represented by *Ramicandelaber fabisporus*.

In Phu Pha Phet Cave, tourists enter and exit from areas A and B, respectively. Karst cave fungi belonging to the phyla Ascomycota and Basidiomycota and including genera such as *Auxarthron, Deconica, Diaporthe, Humicola, Sarocladium,* and *Xylaria* were found in Zone C. This area is separate from the main hall, and tourists are not allowed to enter it. Additionally, cave fungi in Ascomycota, Basidiomycota, and Zoopagomycota, such as *Peroneutypa, Ramicandelaber*, *Acremonium, Fusarium, Gliomastix*, and *Ovicillium*, and especially the species *Ramicandelaber fabisporus* (Zoopagomycota), were only found in Zone C.

**Figure 8:**
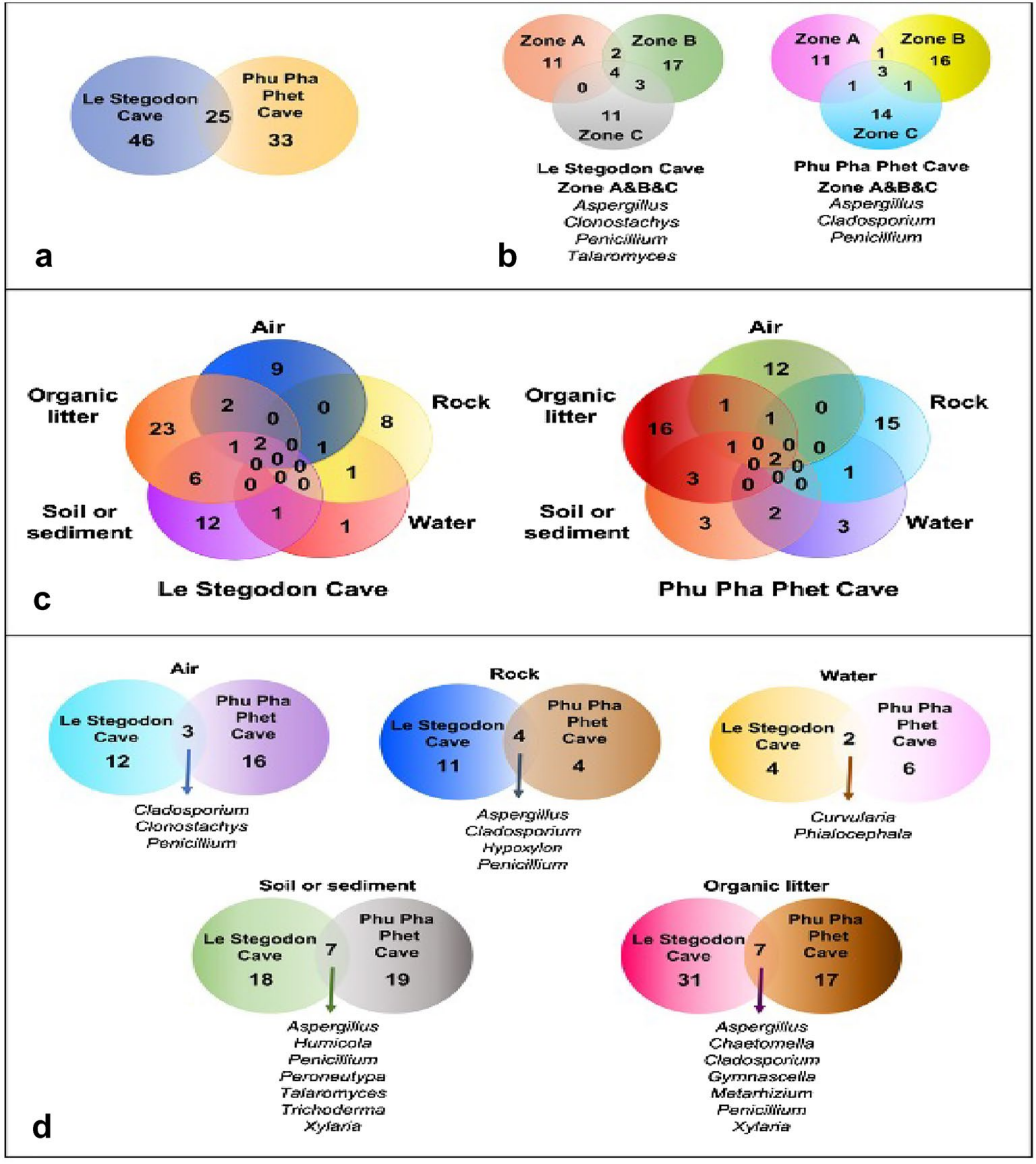
Venn diagrams of the fungal genera found in the two caves. (**a**) The number of fungal genera found in each individual cave and the number found in both caves. (**b**) The number of overlapping and nonoverlapping fungal genera in each zone (A, B and C) of the two caves. (**c**) The numbers of overlapping and exclusive genera in each type of sample collected from the two caves. (**d**) Comparison of the fungal genera found in each type of sample (air, rock, water, soil/sediment, and organic litter) in the two caves.

The fungi from rock, air, water, soil/sediment and organic litter samples in two karst caves were classified at the genus level. In Le Stegodon Cave, the most common rock genera were *Xylaria* (23%), *Nemania* (14%) and *Cladosporium* (10%), while *Penicillium* (17%) dominated the air samples, followed by *Cladosporium* (11%). In water samples, the dominant genera were *Hypoxylon, Nigrograna, Metarhizium* and *Phialocephala* (17%), and in soil/sediment samples, it was *Penicillium* (26%). The organic litter samples were dominated by *Penicillium* (11%), *Aspergillus* (6%) and *Cladosporium* (6%), along with several other genera (Fig. 8).

In Phu Pha Phet Cave, *Cladosporium* (56%) was the most common rock genus, while *Penicillium* (25%) dominated the air samples, and *Aspergillus* (21%) was the most common genus in soil/sediment samples. *Aspergillus, Cladosporium* and *Penicillium* were the most abundant genera in organic litter, comprising 33%, 11% and 8%, respectively (Fig. 8).

Figure 8 compares fungal genera in Le Stegodon Cave and Phu Pha Phet Cave based on three zones and five types of samples. Of the total genera found, 46 were exclusive to Le Stegodon Cave and 33 to Phu Pha Phet Cave, with 25 genera common to both caves. Four genera (*Aspergillus, Clonostachys, Penicillium,* and *Talaromyces*) were present in all zones of Le Stegodon Cave, while three genera (*Aspergillus, Cladosporium,* and *Penicillium*) were present in all zones of Phu Pha Phet Cave. There was no overlap of fungal genera in air and rock samples from both caves (Fig. 8c). Comparing the overlapping genera in the five sample types showed that *Cladosporium, Clonostachys*, and *Penicillium* overlapped in air samples from both caves. *Aspergillus, Cladosporium, Hypoxylon*, and *Penicillium* overlapped in rock samples, while *Curvularia* and *Phialocephala* overlapped in water samples (Fig. 8d).

The results presented in Fig. 8d show that in soil/sediment samples, seven fungal genera were common to both Le Stegodon Cave and Phu Pha Phet Cave. When the organic litter samples from the two caves were compared, it was found that of the 31 fungal genera identified in Le Stegodon Cave and the 17 fungal genera identified in Phu Pha Phet Cave, 7 were found in both caves. Despite the presence of 7 fungal genera in common in the soil/sediment samples from the two caves, 18 fungal genera were identified exclusively in Le Stegodon Cave, and 19 fungal genera were found only in Phu Pha Phet Cave (Fig. 8d).

### Fungal diversity analysis

The diversity indices of the different samples are given in Table 1. Because Shannon’s diversity index correlates with all other diversity indices (Fig. S8), the comparison of per-sample diversity among different types of samples is presented based only on this index. There was a significant effect of sample type on the variation in per-sample diversity (F = 28.83, *p* value = 0.006), while no difference in per-sample diversity was detected between caves (F = 0.375, *p* value = 0.563), between zones (F = 0.417, *p* value = 0.677), or in the effect of the interaction between these factors (Cave: Type, F = 0.32, *p* value = 0.811; Cave: Zone, F = 1.62, *p* value = 0.274; Type: Zone, F = 1.181, *p* value = 0.423).

**Table 1:** Average Shannon’s diversity (H) of the samples of different types in the two studied caves.

Cave	Sample type	H (mean ± sd.)	N
Phu Pha Phet	Air^ab^	2.891 ± 0.314	3
	Organic litters^a^	2.482 ± 0.513	10
	Rock^ab^	3.088 ± 0.234	3
	Soil^b^	3.613 ± 0.365	3
	Water^a^	2.202 ± 0.414	3
Le Stegodon	Air^ab^	3.035 ± 0.165	3
	Organic litters^b^	3.137 ± 0.599	10
	Rock^b^	3.257 ± 0.466	3
	Soil^b^	3.715 ± 0.611	3
	Water^a^	2.078 ± 0.118	3

sd.: Standard deviation, N: Number of samples. The letters in superscript are grouping symbols. The presence of at least one shared grouping symbol means that a significant difference was not detected (*p*value > 0.05). Values followed by the same letter(s) in each column are not significantly different. (^a^ = XX; ^b^ = XX; ^ab^ = XX??).

**Figure 9:**
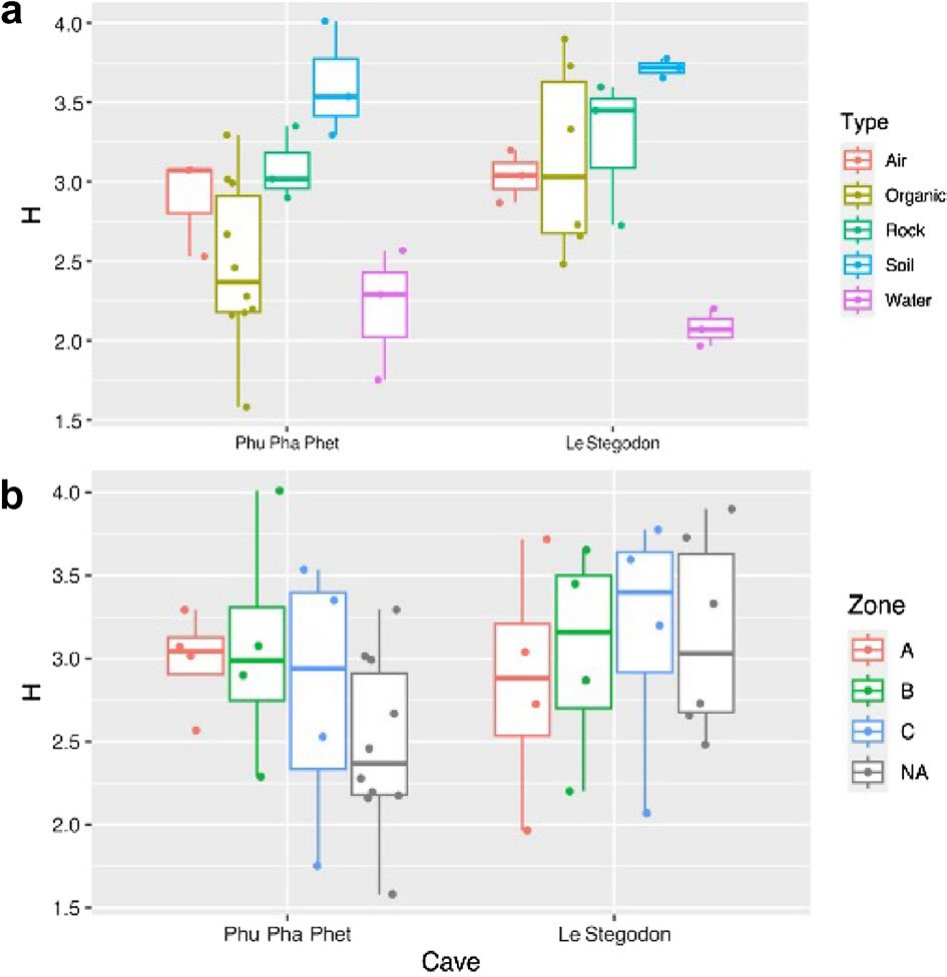
Boxplots showing data points representing the distributions of the per-sample Shannon’s diversity index (H) according to sample type (top panel, **a**) and sampling zone (bottom panel, **b**) within Phu Pha Phet Cave and Le Stegodon Cave. NA = Samples from organic litter for which the sampling zone was not specified.

Per-sample Shannon diversity was highest for soil samples and lowest for water samples in both caves (Fig. 9 and Table 1). We used a two-sided pairwise t test with Bonferroni correction to compare diversity among sample types within each cave. In Phu Pha Phet Cave, significant differences in diversity were found only between the soil samples and the water samples (*p*value = 0.011) and between the soil samples and the organic litter samples (2.482 ± 0.513) (*p* value = 0.011) (Table 1). In Le Stegodon Cave, there were significant differences in diversity only between the water samples and the soil samples (*p* value = 0.004), the rock samples (*p* value = 0.046), and the organic litter samples (*p* value = 0.036) (Table 1).

**Figure 10:**
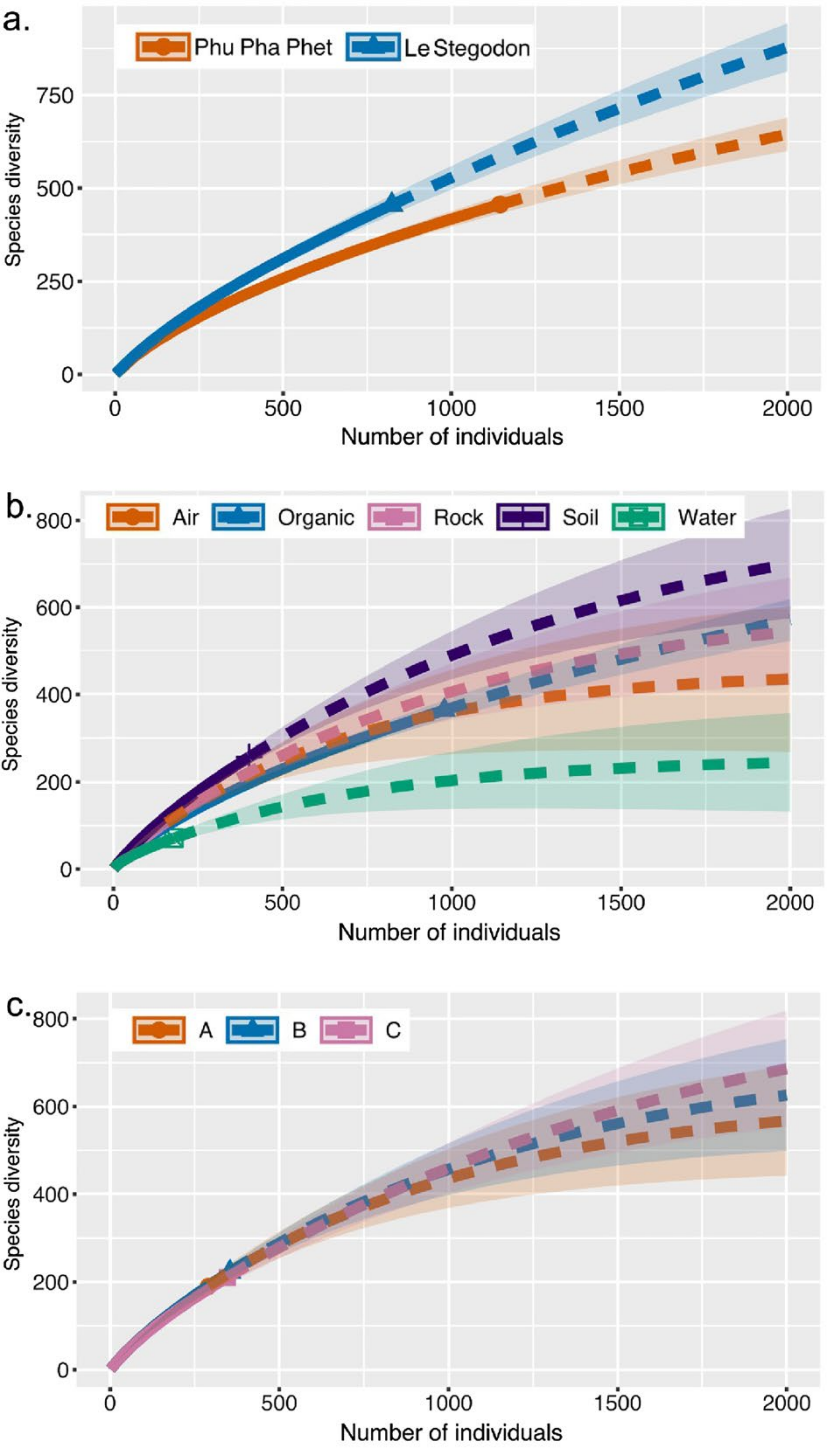
Rarefaction curves and extrapolation curves for (**a**) the two studied caves, (**b**) the sample types as defined in this study, and (**c**) the sampling zones within the caves. The number of individuals is equal to the number of colonies. The unbroken lines represent the curves constructed from the data obtained in the study. The dashed lines represent the corresponding extrapolations.

Table 2 shows similar species richnesses for Phu Pha Phet Cave (456) and Le Stegodon Cave (455), but rarefied species richness was higher for Le Stegodon Cave (455.0) than for Phu Pha Phet Cave (365.647) due to the greater number of colonies counted. The species accumulation curve showed equal numbers of sampled isolates, with Le Stegodon Cave having a higher species richness (Fig. 9). Organic litter samples had the most species (364), followed by soil (257), rock (157), air (106), and water (71). However, rarefied species richness was highest for soil samples (121.066), as indicated by the higher rarefaction curve (Fig. 10). Zone B had the highest species richness (223), followed by Zone C (210) and Zone A (190), while all three zones had equivalent rarefied species richness (Table 2 and Figs. 9, 10).

**Table 2:** Species richness (S), rarefied species richness (S.rare) and Shannon’s diversity index (H) for the caves, sample types, and sampling zones.

Category	S	S.rare	H	N
Cave				
Phu Pha Phet	456	365.647	5.42	1145
Le Stegodon	455	455	5.692	823
Sample type				
Air	106	106.000	4.457	156
Organic litter	364	98.01	5.118	978
Rock	157	107.102	4.745	254
Soil	257	121.066	5.262	401
Water	71	64.323	3.572	179
Sampling zone				
A	190	190	4.951	290
B	223	190.9174	5.086	354
C	210	183.254	4.994	346

N: Number of colonies counted.

## Discussion

The findings on the distribution of fungi in the Satun UNESCO Global Geopark suggest that the park exhibits high fungal diversity compared with other UNESCO World Cultural Heritage sites that are not Global Geoparks, such as the Maijishan Grottoes in China ^33^, Jantar Mantar in India^34^, Sassi in Italy^35^, the old cathedral of Coimbra in Portugal^36^, Lascaux Cave in France^37^, and the Naracoorte Caves in Australia^38^. This study of two caves in the Satun UNESCO Global Geopark found a diverse range of cave fungi, which is consistent with previous studies^6,8^. The Ascomycota was found to be the most common in the studied caves, with a greater diversity of members compared to the Basidiomycota and Zoopagomycota. This supports the findings of Zhang et al.^5^ who reported a higher number of Ascomycota species compared to Basidiomycota species. The fungal genera found in Thai karst caves, such as *Aspergillus, Fusarium, Penicillium*, and *Trichoderma*, are similar to those discovered in Chinese karst caves^5,12^. Adetutu et al.^38^ reported that the Ascomycota was the most prevalent in the Naracoorte Caves in Australia. In addition, Ascomycota has also been found in other locations, including Malaysia^13^, Slovakia^39,40^, and Puerto Rico^41^.

The fungal genera identified in this study are commonly found in other cave habitats, such as *Penicillium* and *Aspergillus*, which have unique characteristics allowing them to grow in diverse environments worldwide^42^. Out et al.^43^ reported 272 fungal isolates from soil samples in the Nakimu Caves of Glacier National Park, Canada, while Nováková^39^ evaluated 195 microfungal species from 73 genera in samples from Slovak Karst National Park in Slovakia. At Shuanghe National Geographic Park in China, 510 fungal isolates were reported^25^. Brad et al.^44^ uncovered 50 culturable filamentous fungi from the Romanian Scărișoara Ice Cave. This study compared the fungal diversity in samples obtained from rock (46 species in 19 genera), air (50 species in 31 genera), seeping water (12 species in 14 genera), soil (44 species in 90 genera), and organic material (55 genera in 108 species). Seeping water samples had lower fungal diversity compared to other sample types, possibly because microbiomes present in the water were trapped in the stalactites' core during seepage through the cave roof to the stalactites.

Ikner et al.^45^ reported that water circulation in caves promotes the growth the proliferation of microorganisms and fungi that produce spores. Their findings parallel our study on two caves visited frequently by tourist groups (Zones A–C), where we assessed the effects of varying degrees of human contact, from high to medium (Zones A–C), and low levels of exposure (Zone C) in this study. The impact of humans on caves can significantly alter the diversity of microbes and fungi^46^. When visitors enter, they can introduce new fungi from their skin, clothing, and breath. Their presence can also cause physical disturbances, changing airflow and disrupting sediments^47,48^. Additionally, the organic matter they bring, such as skin flakes or food particles, can provide nutrients that favor certain microbial growth. Furthermore, artificial lighting used during tours can promote the growth of photosynthetic organisms that normally wouldn't thrive in the dark cave environment. These changes are crucial to understand because microbes and fungi play vital roles in cave ecosystems and their formations^47,48^.

In Le Stegodon Cave, Sordariomycetes was common in rock and water samples, while Dothideomycetes was common in air and organic material samples. Eurotiomycetes was prevalent in soil samples. In Phu Pha Phet Cave, Dothideomycetes was the predominant fungal group in rock and water samples, followed by Eurotiomycetes and Sordariomycetes. These findings align with a study by Ruibal et al.^24^, identifying Dothideomycetes as a significant fungal group in rock and water samples from Spanish caves. This suggests that Dothideomycetes is frequently found in cave environments.

The Microascales was the most common in organic materials from both caves, and it is related to the presence of dung from bats, snakes, arthropods, and snails, which provides nutrients ideal for cave fungi proliferation^39,49^. In five guano samples from Le Stegodon and Phu Pha Phet Caves, we found 20 species representing 16 genera, mainly asexual morphs of *Aspergillus*, *Cladosporium*, and *Penicillium*. Our results align with studies of Malaysian guano^13^ and Puerto Rican caves^41^, which identified several genera such as *Acremonium,* and *Scytalidium.* Mucoromycota was found in Le Stegodon Cave, categorized into 7 genera and 8 species of Mucoromycetes, Mortierellomycetes, and Umbelopsidomycetes. *Mortierella* and *Mucor* are common in soil and organic matter in Nakimu caves according to Out et al.^43^. Oligotrophic fungi in Phu Pha Phet Cave's organic debris were identified as *Cephalotrichum purpureofuscum* and three novel fungal species of the genus *Cephalotrichum* in China^25^. In non-tourist areas of the caves, three yeast species representing *Glactomyces* sp. and an unclassified species in the order Saccharomycetales were isolated. Our culturable fungi are highly diverse and known to act as endophytic and pathogenic fungi.

Among the factors that affect the abundance of fungi in karst caves, most fungal species found inside caves originate from the outside cave environment because of airflow and traffic by humans and animals^12,50^. Fungal multiplicity is relatively high near cave entrance zones and decreases with depth, according to Ogórek et al.^51^. This fungus has also been discovered in caves in Romania^52^ and China^5^. In our study, we found various entomopathogenic fungi that may represent potential biocontrol agents, including *Lecanicillium* sp., *Metarhizium marquandii* [Current Name: *Marquandomyces marquandii*; Mongkolsamrit et al. 2020], *Metarhizium*, and *Purpureocillium*. These fungi have previously been employed for biological control^53^, and *Trichoderma harzianum* was identified as a plant pathogenic fungus isolated from soil and organic matter.

Fungal diversity in karst caves can reveal the ecological balance and overall health of these distinctive habitats. These caves have low temperatures, high humidity, a lack of nutrients, and darkness, making fungi a vital part of their ecosystem. Fungi play crucial roles in nutrient cycling, decomposition, and interactions with other organisms. Therefore, studying fungal diversity and identifying new fungal taxa in the dark zones of karst caves can provide insight into their functioning and the impact of human activities on these environments^46^.

Our diversity analysis suggests that the difference in species found in Phu Pha Phet Cave, Le Stegodon Cave, and organic litter samples may be due to sampling limitations. Soil samples had the highest species richness based on rarefaction analysis, likely due to optimal growth conditions, essential nutrients, moisture levels, and physical structure. Soil's nutrient-rich environment, stable physical structure, and sheltered conditions make it an ideal habitat for fungal communities, contributing to its perception as a diverse fungal habitat^54^. Le Stegodon Cave is expected to have more species than Phu Pha Phet Cave due to the presence of dual exits, facilitating greater exchange of air, light, and organic matter with the surrounding environment, resulting in more diverse habitats for fungal growth. This diversity supports a more diverse community of fungi, thus increasing fungal diversity in the cave. In contrast, caves such as Phu Pha Phet Cave with only a single exit have limited resources for fungal growth, resulting in lower fungal diversity.

Sample type is the primary factor that affects the fungal community's species diversity and community structure in cave habitats. Random sampling may yield similar diversity in samples from Phu Pha Phet Cave and Le Stegodon Cave. However, the overall higher diversity in Le Stegodon Cave suggests that species rarefaction could be due to its connection to the sea via its western outlet. Further research on the diversity of marine fungi near Le Stegodon Cave is necessary to test this hypothesis. Conversely, the location within the cave (Zone A, B, or C) did not appear to impact fungal diversity or community structure. This observation is consistent with recent research from a publication that documented the identification of four new species within Le Stegodon Cave^55^.

Fungi in karst caves have the potential to produce biologically active compounds, making it crucial to study their diversity. Understanding the mycological richness and habitat of the UNESCO Satun Cave can aid our understanding of these environments and promote sustainable conservation and exploitation. This knowledge would benefit future planning, monitoring, and management in Thailand aimed at balancing conservation, development, and exploitation. To further protect the cave environment and understand the role of fungal species and mycobiota in cave ecosystems' evolution, it is essential to identify which fungal communities reside in the cave and how their metabolic activities affect the cave environment. Omics technologies such as DNA metabarcoding are expected to enhance our understanding of the functional diversity within cave microbiomes^46,54^. This knowledge is crucial in evaluating the impact of human activities such as tourism on these ecosystems and in designing effective management plans. Furthermore, it will aid in identifying appropriate methods for restoring cave microbiomes in areas of the Satun UNESCO Global Geopark that have been disturbed by tourism^38,52^.

### Supplementary Information

Supplementary Information.

## Data Availability

Sequence data that support the findings of this study have been deposited in GenBank with the accession numbers (the 319 fungal culturable isolates based on ITS data): OR077530–OR077579, OR081370–OR081419, OR095903–OR095959, OR095960–OR096009, OR096308–OR096357 and OR122849–OR122898.

## References

[1] Schoch CL (2009). The Ascomycota tree of life: A phylum-wide phylogeny clarifies the origin and evolution of fundamental reproductive and ecological traits. Syst. Biol..

[2] Bastian F, Jurado V, Nováková A, Alabouvette C, Saiz-Jimenez C (2010). The microbiology of Lascaux Cave. Microbiology.

[3] Naranjo-Ortiz MA, Gabaldón T (2019). Fungal evolution: Major ecological adaptations and evolutionary transitions. Biol. Rev..

[4] Hawksworth DL, Lücking R (2017). Fungal diversity revisited: 2.2 to 3.8 million species. Microbiol. Spectr..

[5] Zhang ZF (2017). Culturable mycobiota from Karst caves in China, with descriptions of 20 new species. Persoonia.

[6] Zhang Z-F, Zhao P, Cai L (2018). Origin of cave fungi. Front. Microbiol..

[7] Zhu H-Z, Jiang C-Y, Liu S-J (2022). Microbial roles in cave biogeochemical cycling. Front. Microbiol..

[8] Vanderwolf K, Malloch D, McAlpine D, Forbes G (2013). A world review of fungi, yeasts, and slime molds in caves. Inter. J. Speleol..

[9] Fernández-Remacha D (2022). Analysis of laccase-like enzymes secreted by fungi isolated from a cave in northern Spain. MicrobiologyOpen.

[10] Gubiani JR (2022). Absolute configuration of cytotoxic anthraquinones from a Brazilian cave soil-derived fungus, Aspergillus sp. SDC28. Archiv. der Pharmazie.

[11] Cunha AOB (2020). Living in the dark: Bat caves as hotspots of fungal diversity. Plos One.

[12] Zhang Z-F (2021). Culturable mycobiota from Karst caves in China II, with descriptions of 33 new species. Fungal Divers..

[13] Wasti IG (2021). Fungal communities in bat guano, speleothem surfaces, and cavern water in Madai cave, Northern Borneo (Malaysia). Mycology.

[14] Karunarathna SC (2020). Discovery of novel fungal species and pathogens on bat carcasses in a cave in Yunnan Province, China. Emerg. Microbes Infect..

[15] Pereira MLS (2022). Richness of *Cladosporium* in a tropical bat cave with the description of two new species. Mycol. Prog..

[16] Leplat J, Alexandre F, Bousta F (2022). *Leptobacillium cavernicola*, a newly discovered fungal species isolated from several Paleolithic-decorated caves in France. Phytotaxa.

[17] Visagie CM, Goodwell M, Nkwe DO (2021). *Aspergillus* diversity from the Gcwihaba Cave in Botswana and description of one new species. Fungal Syst. Evol..

[18] Nuankaew S (2022). Two novel species of *Talaromyces* discovered in a karst cave in the Satun UNESCO Global Geopark of southern Thailand. J. Fungi..

[19] Cheablam O, Tansakul P, Nantakat B, Pantaruk S (2021). Correction to: Assessment of the geotourism resource potential of the Satun UNESCO Global Geopark, Thailand. Geoheritage.

[20] Nantakat B, Vorachart V (2021). Designing tourism identity communication in Satun UNESCO Global Geopark. GeoJ Tour. Geosites.

[21] Siripattharapurinont, R. *Official report of cave survey and mapping for Phu Pha Phet Cave.*, <http://www.oic.go.th/FILEWEB/CABINFOCENTER6/DRAWER013/GENERAL/DATA0000/00000243.PDF> (2015).

[22] Siripattharapurinont, R. *Official report of cave survey and mapping for Le Stegodon Cave.*, <http://www.oic.go.th/FILEWEB/CABINFOCENTER6/DRAWER013/GENERAL/DATA0000/00000242.PDF> (2016).

[23] Muangsong C, Cai B, Pumijumnong N, Lei G, Wang F (2019). A preliminary study on teak tree ring cellulose δ18O from northwestern Thailand: the potential for developing multiproxy records of Thailand summer monsoon variability. Theor. Appl. Climatol..

[24] Ruibal C, Platas G, Bills GF (2005). Isolation and characterization of melanized fungi from limestone formations in Mallorca. Mycol Prog.

[25] Jiang J-R, Cai L, Liu F (2017). Oligotrophic fungi from a carbonate cave, with three new species of *Cephalotrichum*. Mycology.

[26] Ogórek R, Dyląg M, Višňovská Z, Tancinová D, Zalewski D (2016). Speleomycology of air and rock surfaces in Driny Cave (Lesser Carpathians, Slovakia). J. Cave Karst Stud..

[27] Boonyuen N (2021). Novelties in Fuscosporellaceae (Fuscosporellales): Two new *Parafuscosporella* from Thailand revealed by morphology and phylogenetic analyses. Diversity.

[28] White, T. J., Brun, T., Lee, S. & Taylor, J. W. in *PCR protocols: A guide to methods and applications* (eds M. A. Innis, D. H. Gelfand, J. J. Sninsky, & T. J. White) Ch. 315–322, 482 (Academic, 1990).

[29] Hall TA (1999). BioEdit: a user-friendly biological sequence alignment editor and analysis program for Windows 95/98/NT. Nucleic Acids Symp. Ser..

[30] Oksanen, J. *et al. vegan community ecology package version 2.6–4*, <https://cran.r-project.org/web/packages/vegan/index.html> (2022).

[31] Chao A (2014). Rarefaction and extrapolation with Hill numbers: A framework for sampling and estimation in species diversity studies. Ecol. Monogr..

[32] Hsieh TC, Ma K, Chao A (2016). iNEXT: An R package for rarefaction and extrapolation of species diversity (Hill numbers). Methods Ecol. Evol..

[33] He D (2021). Insights into the bacterial and fungal communities and microbiome that causes a microbe outbreak on ancient wall paintings in the Maijishan Grottoes. Int. Biodeterior. Biodegrad..

[34] Yadav M, Gupta V (2021). Assessment of microbial diversity and their role in deterioration of Jantar-Mantar, Jaipur, India. Indian J. Ecol..

[35] Mang SM, Scrano L, Camele I (2020). Preliminary studies on fungal contamination of two Rupestrian Churches from Matera (Southern Italy). Sustainability.

[36] Trovão J (2019). Fungal diversity and distribution across distinct biodeterioration phenomena in limestone walls of the old cathedral of Coimbra, UNESCO World Heritage site. Int. Biodeterior. Biodegrad..

[37] Martin-Sanchez PM, Bastian F, Alabouvette C, Saiz-Jimenez C (2013). Real-time PCR detection of *Ochroconis lascauxensis* involved in the formation of black stains in the Lascaux Cave France. Sci. Total Environ..

[38] Adetutu EM (2011). Phylogenetic diversity of fungal communities in areas accessible and not accessible to tourists in Naracoorte Caves. Mycologia.

[39] Novakova A (2009). Microscopic fungi isolated from the Domica Cave system (Slovak Karst National Park, Slovakia) A review. Inter. J. Speleol..

[40] Ogórek R, Kozak B, Višňovská Z, Tančinová D (2018). Phenotypic and genotypic diversity of airborne fungal spores in Demänovská Ice Cave (Low Tatras, Slovakia). Aerobiologia (Bologna).

[41] Nieves-Rivera ÁM, Santos C, Dugan F, Miller TE (2009). Guanophilic fungi in three caves of southwestern Puerto Rico. Inter. J. Speleol..

[42] Houbraken, J., de Vries, R. P. & Samson, R. A. in *Adv. Appl. Microbiol.* Vol. 86 (eds Sima Sariaslani & Geoffrey M. Gadd) 199–249 (Academic Press, 2014). 10.1016/B978-0-12-800262-9.00004-4 24377856

[43] Out B, Boyle S, Cheeptham N (2016). Identification of fungi from soil in the Nakimu caves of Glacier National park. J. Exper. Microbiol. Immunol..

[44] Brad T (2018). Fungi in perennial ice from Scărișoara Ice Cave (Romania). Sci. Rep..

[45] Ikner LA (2007). Culturable microbial diversity and the impact of tourism in Kartchner Caverns. Arizona. Microb. Ecol..

[46] Bontemps Z, Hugoni M, Moënne-Loccoz Y (2023). Microscale dynamics of dark zone alterations in anthropized karstic cave shows abrupt microbial community switch. Sci. Total Environ..

[47] Bontemps Z, Alonso L, Pommier T, Hugoni M, Moënne-Loccoz Y (2022). Microbial ecology of tourist Paleolithic caves. Sci. Total Environ..

[48] Bercea S, Năstase-Bucur R, Moldovan OT, Kenesz M, Constantin S (2019). Yearly microbial cycle of human exposed surfaces in show caves. Subterr. Biol..

[49] Jurado V (2010). Fungal outbreak in a show cave. Sci. Total Environ..

[50] Dominguez-Moñino I, Jurado V, Rogerio-Candelera MA, Hermosin B, Saiz-Jimenez C (2021). Airborne fungi in show caves from Southern Spain. Appl. Sci..

[51] Ogórek R, Lejman A, Matkowski K (2013). Fungi isolated from Niedźwiedzia Cave in Kletno (Lower Silesia, Poland). Inter. J. Speleol..

[52] Novakova A, Kubátová A, Sklenář F, Hubka V (2018). Microscopic fungi on cadavers and skeletons from cave and mine environments. Czech Mycol..

[53] Subramaniam MSR, Babu A, Deka B (2021). *Lecanicillium lecanii* (Zimmermann) Zare & Gams, as an efficient biocontrol agent of tea thrips, *Scirtothrips bispinosus* Bagnall (Thysanoptera: Thripidae). Egypt. J. Biol. Pest Control.

[54] Martin-Pozas T (2022). Diversity of microfungi in a High Radon Cave ecosystem. Front. Microbiol..

[55] Preedanon S (2023). Eight novel cave fungi in Thailand's Satun Geopark. Fungal Syst. Evol..

