# Supplementary material for: Unravelling the hidden diversity of cave mycobiota in Thailand’s Satun Geopark
Source: Sci Rep. 2023 Nov 6;13:19162. doi: 10.1038/s41598-023-43316-2 (PMC10628224; doi:10.1038/s41598-023-43316-2)
Supplement: Supplementary file 1 — Supplementary Information. [file 41598_2023_43316_MOESM1_ESM.docx]

**Supplemental Tables**

**Table S1.** Physical properties of the cave environments and water samples from Phu Pha Phet and Le Stegodon Caves.

**Table S2.** The six most common genera obtained from two karst caves.

**Supplementary Figures**

**Figure S1** The most common classes of fungi (*Eurotiomycetes, Sordariomycetes* and *Dothideomycetes*) found in Le Stegodon Cave (upper portion of the figure) and Phu Pha Phet Cave (lower portion of the figure).

**Figure S2** Percentages of fungal isolates identified as belonging to the fungal classes recorded in Le Stegodon Cave according to the type of sample (rock, air, water, soil/sediment and organic litter) in which they were found.

**Figure S3.** Percentages of fungal isolates identified as belonging to the fungal classes recorded in in Phu Pha Phet Cave according to the type of sample (rock, air, water, soil/sediment and organic litter) in which they were found.

**Figure S4.** Percentages of fungal isolates identified as belonging to the fungal orders recorded in Le Stegodon Cave according to the type of sample (rock, air, water, soil/sediment and organic litter) in which they were found.

**Figure S5.** Percentages of fungal isolates identified at the order level in Phu Pha Phet Cave according to the type of sample (rock, air, water, soil/sediment and organic litter) in which they were found.

**Figure S6.** Percentages of fungal isolates identified at the genus level in each of the three zones of Le Stegodon Cave.

**Figure S7.** Percentages of fungal isolates identified at the genus level in each of the three zones of Phu Pha Phet Cave.

**Figure S8.** Correlations between Shannon’s diversity index (H) and a.) Simpson’s diversity index (D), b.) species richness (S), and c.) rarefied species richness (S.rare).


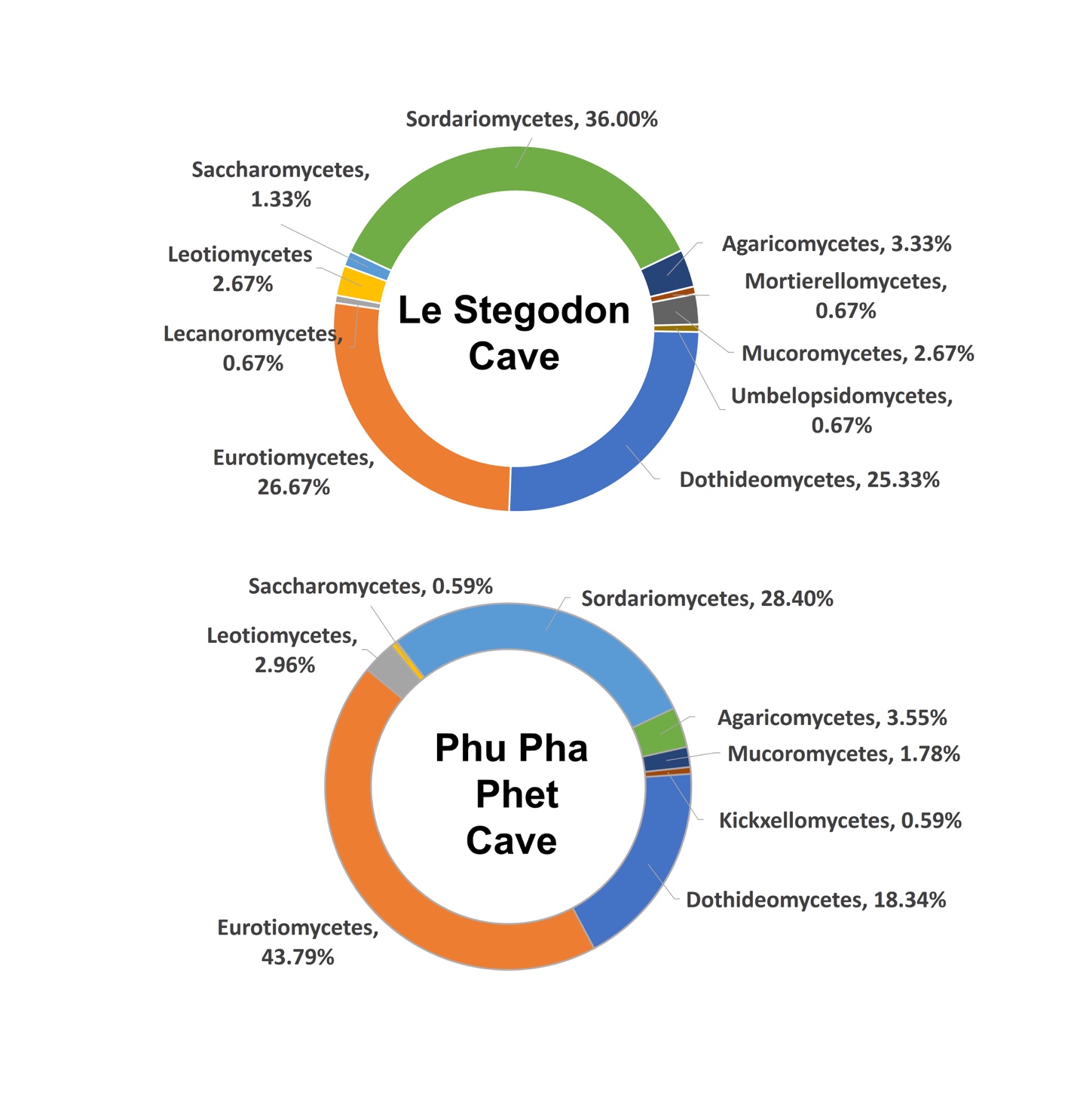


**Figure S1.** The most common classes of fungi (*Eurotiomycetes, Sordariomycetes* and *Dothideomycetes*) found in Le Stegodon Cave (upper portion of the figure) and Phu Pha Phet Cave (lower portion of the figure).


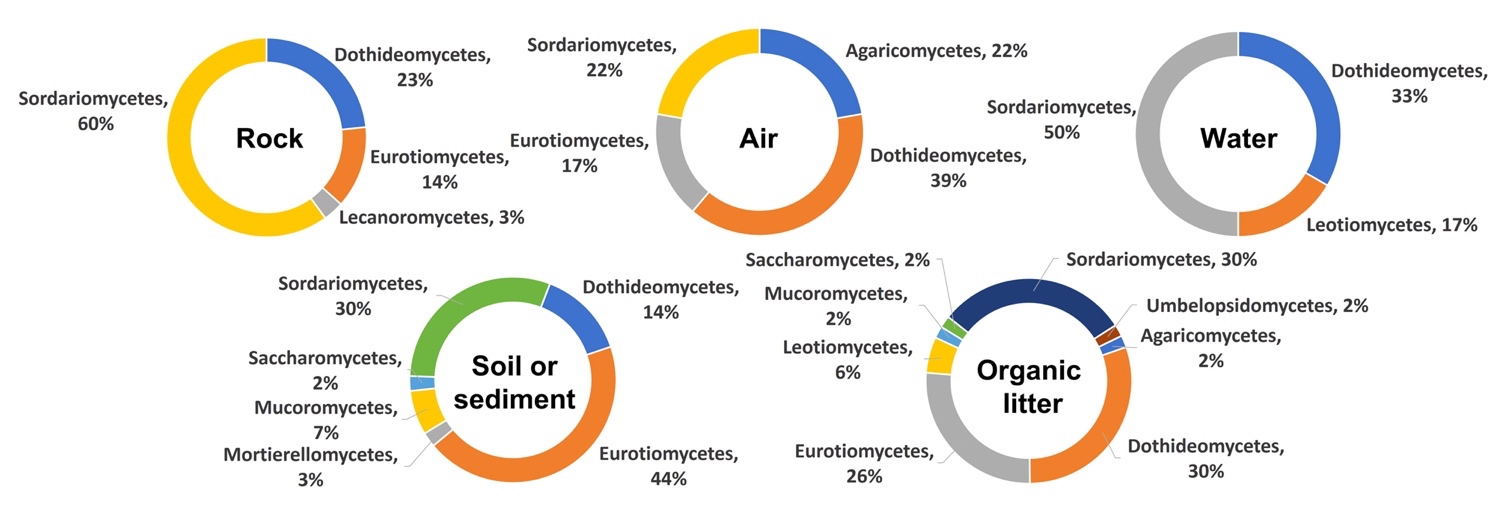


**Figure S2.** Percentages of fungal isolates identified as belonging to the fungal classes recorded in Le Stegodon Cave according to the type of sample (rock, air, water, soil/sediment and organic litter) in which they were found.


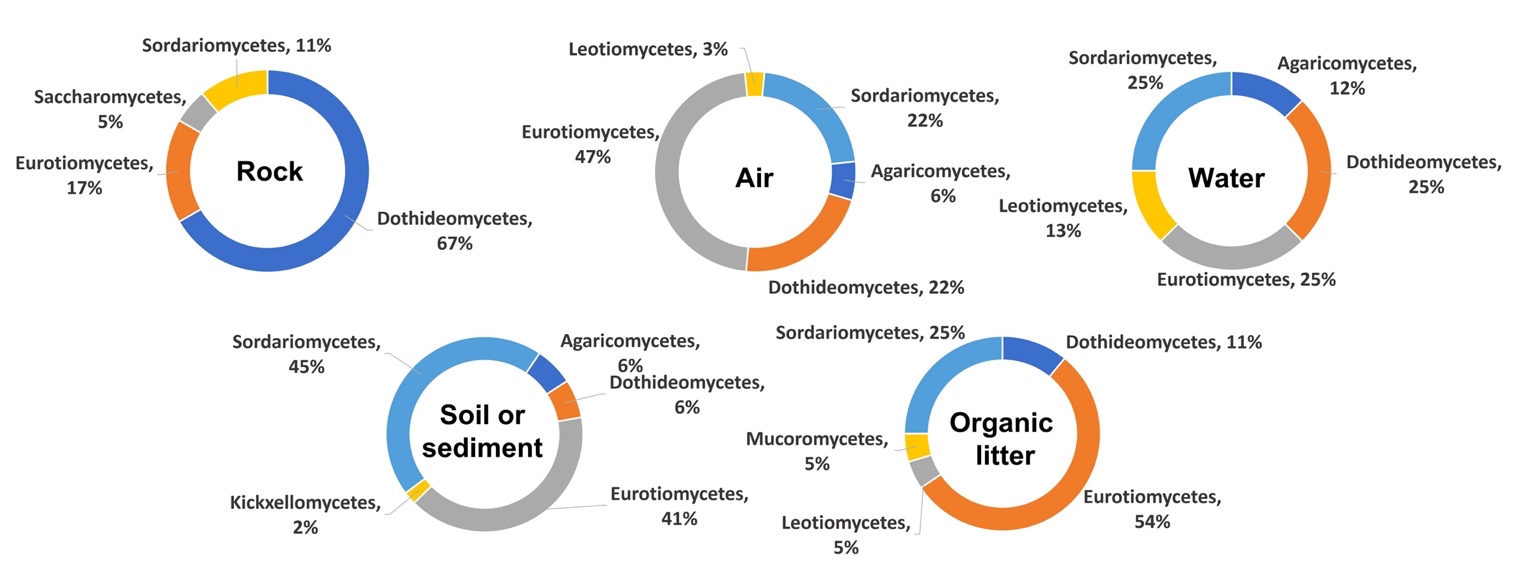


**Figure S3.** Percentages of fungal isolates identified as belonging to the fungal classes recorded in in Phu Pha Phet Cave according to the type of sample (rock, air, water, soil/sediment and organic litter) in which they were found.


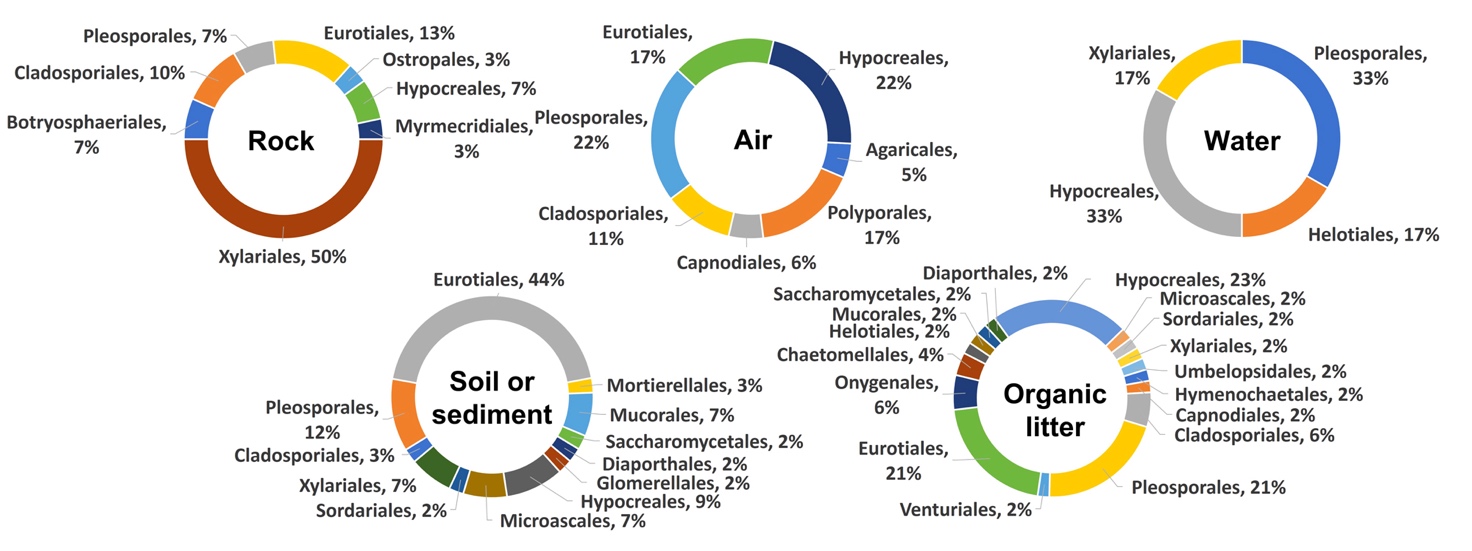


**Figure S4.** Percentages of fungal isolates identified as belonging to the fungal orders recorded in Le Stegodon Cave according to the type of sample (rock, air, water, soil/sediment and organic litter) in which they were found.


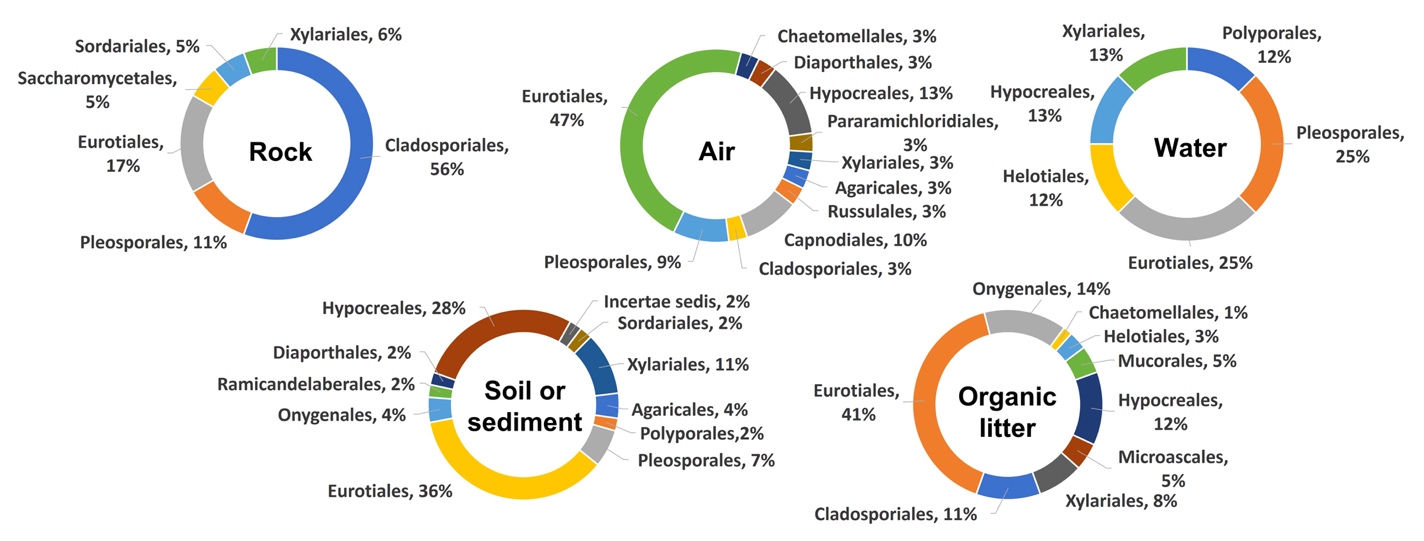


**Figure S5.** Percentages of fungal isolates identified at the order level in Phu Pha Phet Cave according to the type of sample (rock, air, water, soil/sediment and organic litter) in which they were found.


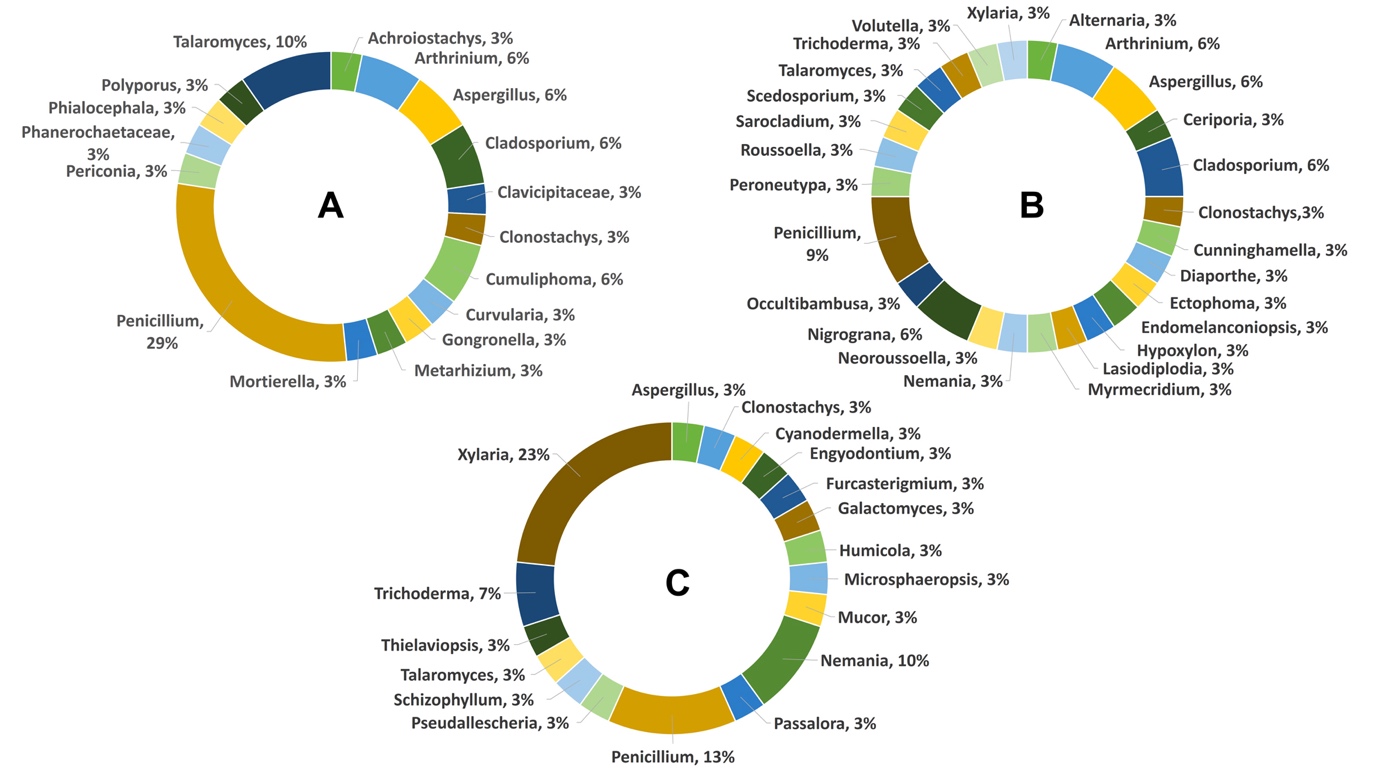


**Figure S6.** Percentages of fungal isolates identified at the genus level in each of the three zones of Le Stegodon Cave.

**
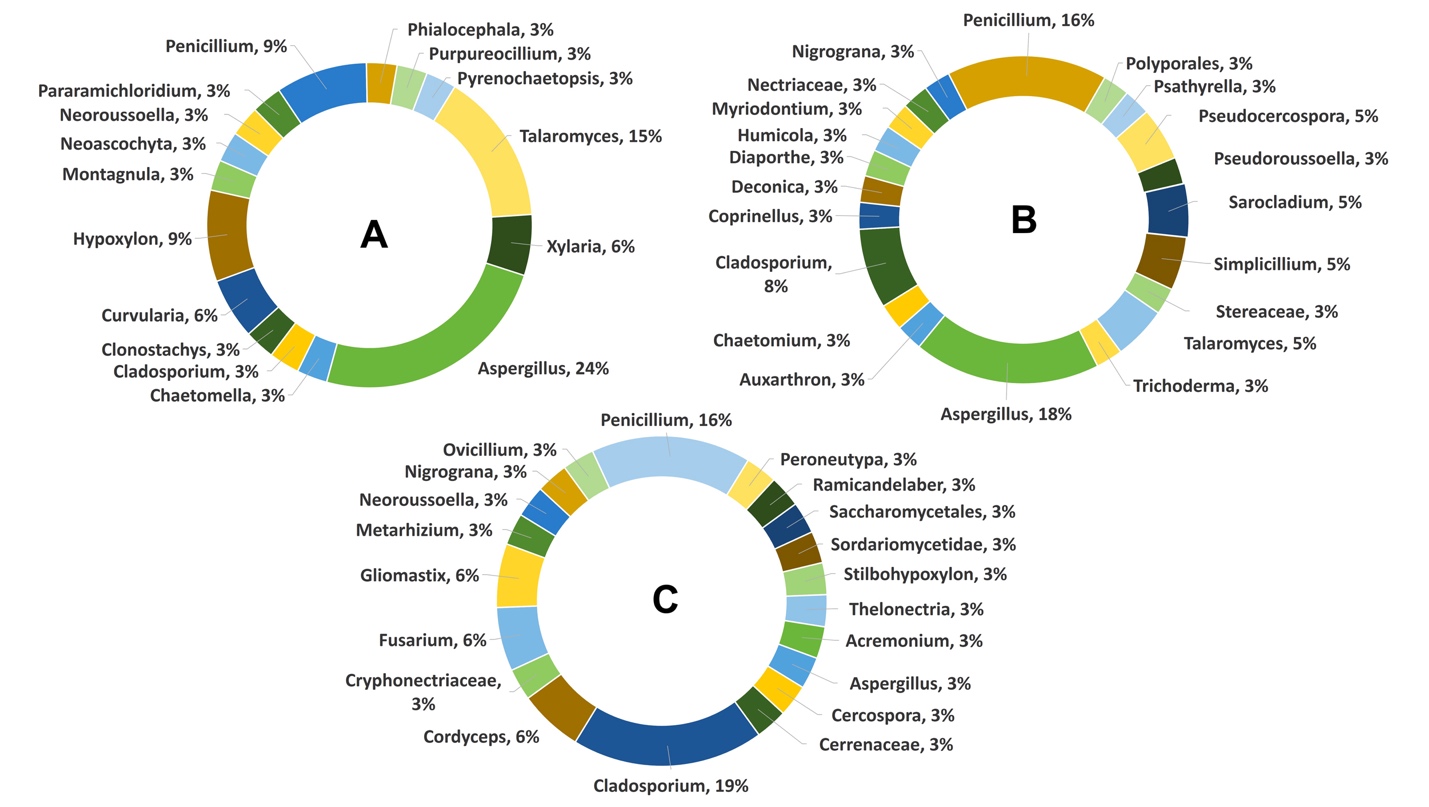
**

**Figure S7.** Percentages of fungal isolates identified at the genus level in each of the three zones of Phu Pha Phet Cave.


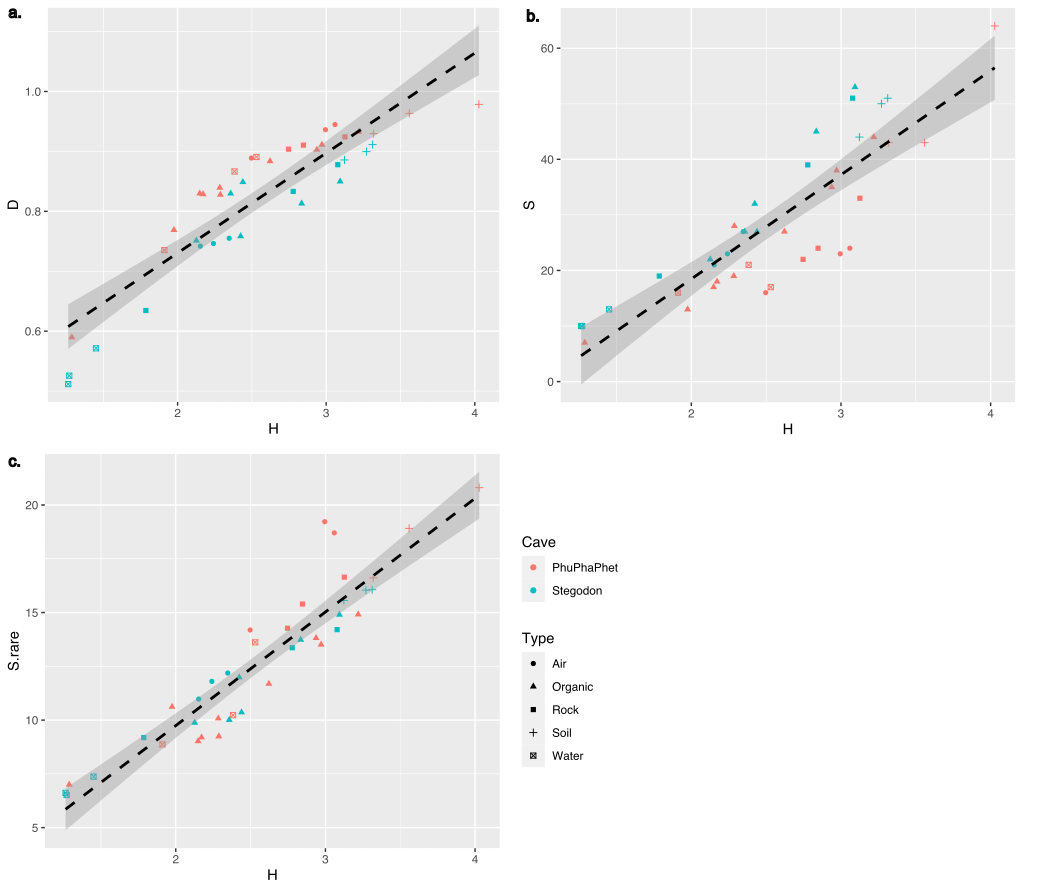


**Figure S8.** Correlations between Shannon’s diversity index (H) and a.) Simpson’s diversity index (D), b.) species richness (S), and c.) rarefied species richness (S.rare).

**Supplemental Tables**

**Table S1.** Physical properties of the cave environments and water samples from Phu Pha Phet and Le Stegodon Caves

| **Phu Pha Phet Cave** | **Zone** | **CO_2_ (ppm)** | **RH (%)** | **Air temp. (°C)** | **Rock surface temp. (°C)** | **Light intensity (Lux)** | **Water sample type** | **pH** | **EC (µS/cm)** | **Salinity (ppt)** | **DO**  **(mg/l)** | **CaCO_3_ concentration (mg/l)** |
| --- | --- | --- | --- | --- | --- | --- | --- | --- | --- | --- | --- | --- |
|  | A1 | 420 | 99.9 | 25.5 | 26.5 | 0 | NA | NA | NA | NA | NA | NA |
|  | A2 | 437 | 99.9 | 24.8 | NA | 0 | NA | NA | NA | NA | NA | NA |
|  | A3 | 407 | 99.9 | 25.1 | 25.5 | 0 | NA | NA | NA | NA | NA | NA |
|  | A4 | 551 | 99.9 | 25.1 | 25.8 | 0 | NA | NA | NA | NA | NA | NA |
|  | A5 | 508 | 99.9 | 25.1 | 25.1 | 173 | NA | NA | NA | NA | NA | NA |
|  | B1 | 418 | 99.9 | 25.0 | 25.4 | 0 | NA | NA | NA | NA | NA | NA |
|  | B2 | 461 | 99.9 | 25.0 | 25.4 | 0 | NA | NA | NA | NA | NA | NA |
|  | B3 | 447 | 99.9 | 24.9 | 23.9 | 0 | NA | NA | NA | NA | NA | NA |
|  | B4 | 455 | 99.9 | 24.7 | 22.7 | 0 | NA | NA | NA | NA | NA | NA |
|  | B5 | 438 | 99.9 | 24.9 | 24.4 | 0 | NA | NA | NA | NA | NA | NA |
|  | C1 | 555 | 99.9 | 25.3 | 24.6 | 0 | Drip water | 6.7 | NA | 2.02 | 123 | 125 |
|  | C2 | 645 | 99.9 | 25.3 | 25.4 | 0 | NA | NA | NA | NA | NA | NA |
|  | C3 | 783 | 99.9 | 24.9 | 24.4 | 0 | Pool water | 6.8 | 108 | 0.12 | 112 | 125 |
|  | C4 | 839 | 99.9 | 25.2 | 24.8 | 0 | Drip water | 6.8 | 107 | 0.09 | 143 | 110 |
|  | C5 | 839 | 99.9 | 25.2 | 24.8 | 0 | Drip water | 6.8 | 107 | 0.09 | 143 | 110 |
| **Le Stegodon Cave** | **Zone** | **CO_2_ (ppm)** | **RH (%)** | **Air temp. (°C)** | **Rock surface temp. (°C)** | **Light intensity (Lux)** | **Water sample type** | **pH** | **EC (µS/cm)** | **Salinity (ppt)** | **DO**  **(mg/l)** | **CaCO_3_ concentration (mg/l)** |
|  | A1 | 444 | 99.9 | 26.2 | 26.9 | 0 | Stream | 7.13 | 157 | 0.15 | 7.1 | 4.1 |
|  | A2 | 467 | 99.9 | 24.7 | 27.4 | 0 | Stream | 7.19 | 157 | 0.15 | 3.7 | 3.7 |
|  | A3 | 568 | 99.9 | 25.7 | 27.9 | 0 | Stream | 7.31 | 155 | 0.15 | 7.1 | 7.1 |
|  | A4 | 590 | 99.9 | 25.9 | 27.7 | 0 | Stream | 7.20 | 159 | 0.15 | 7.9 | 6.7 |
|  | A5 | 592 | 99.9 | 25.8 | 27.8 | 0 | Stream | 7.34 | 153 | 0.15 | 8.5 | 8.5 |
|  | Z2 | 645 | 99.9 | 25.8 | 27.4 | 0 | NA | NA | NA | NA | NA | NA |
|  | B1 & Z5 | 432 | 99.9 | 25.9 | 28.1 | 0 | Stream | 7.63 | 155 | 0.15 | 7.2 | 7.2 |
|  | B2 | 604 | 99.9 | 25.5 | 26.9 | 0 | Stream | 7.64 | 155 | 0.15 | 5.4 | 5.4 |
|  | B3 | 523 | 99.6 | 25.6 | NA | 0 | Stream | 7.59 | 152 | 0.15 | 5.7 | 5.7 |
|  | B4 | 496 | 99.9 | 25.6 | 27.0 | 0 | Stream | 7.63 | 155 | 0.15 | 6.8 | 6.8 |
|  | B5 | 449 | 99.4 | 26.2 | 27.5 | 0 | Stream | 7.64 | 152 | 0.15 | 3.4 | 3.4 |
|  | C1 | 625 | 99.1 | 25.7 | 26.6 | 0 | Stream | 7.73 | 172 | 0.17 | 5.7 | 5.7 |
|  | C2 | 769 | 99.9 | 25.5 | 26.6 | 0 | Stream | 7.58 | 185 | 0.60 | 6.7 | 6.7 |
|  | C3 | 959 | 99.9 | 25.7 | 26.4 | 0 | Stream | 7.55 | NA | 2.00 | 5.4 | 5.4 |
|  | C4 | 1081 | 99.9 | 26.2 | 26.2 | 0 | Stream | 7.33 | NA | 6.3 | 6.4 | 6.4 |
|  | C5 | 1120 | 99.9 | 25.9 | 26.3 | 0 | Stream | 7.17 | NA | 7.2 | 6.1 | 5.9 |

N/A = not applicable; RH = relative humidity; EC = electrical conductivity; DO = dissolved oxygen

**Table S2.** The six most common genera obtained from two karst caves

| **Genus** | **Species)** | **Isolate and Section** | **Types of fungal samples obtained** |
| --- | --- | --- | --- |
| ***Aspergillus*** | **45** | **45 strains, 6 sections** | Organic litter and soil/sediment |
| ***Cladosporium*** | **24** | **27 strains** | Rock and organic litter |
| ***Penicillium*** | **41** | **41 strains, 9 sections** | Soil/sediment, air and organic litter |
| ***Talaromyces*** | **14** | **14 strains, 3 sections** | Soil/sediment |
| ***Trichoderma*** | **6** | **6 strains** | Organic litter and soil/sediment |
| ***Xylaria*** | **14** | **14 strains** | Organic litter and soil/sediment |
